# Physical activity and the risk of gestational diabetes mellitus: a systematic review and dose–response meta-analysis of epidemiological studies

**DOI:** 10.1007/s10654-016-0176-0

**Published:** 2016-08-02

**Authors:** Dagfinn Aune, Abhijit Sen, Tore Henriksen, Ola Didrik Saugstad, Serena Tonstad

**Affiliations:** 1 Department of Epidemiology and Biostatistics, School of Public Health, Imperial College London, St. Mary’s Campus, Norfolk Place, Paddington, London, W2 1PG UK; 2 Department of Public Health and General Practice, Faculty of Medicine, Norwegian University of Science and Technology, Trondheim, Norway; 3 Section of Obstetrics, Division of Obstetrics and Gynaecology, Rikshospitalet, Oslo University Hospital, Oslo, Norway; 4 Department of Pediatric Research, Rikshospitalet, Oslo University Hospital, University of Oslo, Oslo, Norway; 5 Section of Preventive Cardiology, Department of Endocrinology, Morbid Obesity and Preventive Medicine, Oslo University Hospital Ullevål, Oslo, Norway

**Keywords:** Physical activity, Exercise, Walking, Gestational diabetes, Abnormal glucose tolerance, Review, Meta-analysis

## Abstract

Physical activity has been inconsistently associated with risk of gestational diabetes mellitus in epidemiological studies, and questions remain about the strength and shape of the dose–response relationship between the two. We therefore conducted a systematic review and meta-analysis of cohort studies and randomized trials on physical activity and gestational diabetes mellitus. PubMed, Embase and Ovid databases were searched for cohort studies, and randomized controlled trials of physical activity and risk of gestational diabetes mellitus, up to August 5th 2015. Summary relative risks (RRs) were estimated using a random effects model. Twenty-five studies (26 publications) were included. For total physical activity the summary RR for high versus low activity was 0.62 (95 % CI 0.41–0.94, I^2^ = 0 %, *n* = 4) before pregnancy, and 0.66 (95 % CI 0.36–1.21, I^2^ = 0 %, *n* = 3) during pregnancy. For leisure-time physical activity the respective summary RRs for high versus low activity was 0.78 (95 % CI 0.61–1.00, I^2^ = 47 %, *n* = 8) before pregnancy, and it was 0.80 (95 % CI 0.64–1.00, I^2^ = 17 %, *n* = 17) during pregnancy. The summary RR for pre-pregnancy activity was 0.70 (95 % CI 0.49–1.01, I^2^ = 72.6 %, *n* = 3) per increment of 5 h/week and for activity during pregnancy was 0.98 (95 % CI 0.87–1.09, I^2^ = 0 %, *n* = 3) per 5 h/week. There was evidence of a nonlinear association between physical activity before pregnancy and the risk of gestational diabetes mellitus, p_nonlinearity_ = 0.005, with a slightly steeper association at lower levels of activity although further reductions in risk were observed up to 10 h/week. There was also evidence of nonlinearity for physical activity in early pregnancy, p_nonlinearity_ = 0.008, with no further reduction in risk above 8 h/week. There was some indication of inverse associations between walking (before and during pregnancy) and vigorous activity (before pregnancy) and the risk of gestational diabetes mellitus. This meta-analysis suggests that there is a significant inverse association between physical activity before pregnancy and in early pregnancy and the risk of gestational diabetes mellitus. Further studies are needed to clarify the association between specific types and intensities of activity and gestational diabetes mellitus.

**Electronic supplementary material:**

The online version of this article (doi:10.1007/s10654-016-0176-0) contains supplementary material, which is available to authorized users.

## Introduction

Gestational diabetes mellitus is an important cause of maternal and perinatal complications including preeclampsia, gestational hypertension, caesarean section, macrosomia, and stillbirths [1]. Gestational diabetes mellitus is a glucose intolerance discovered for the first time in pregnancy and is by the National Institute for Health and Care Excellence (NICE) defined as a fasting plasma glucose of 5.6 mmol/L or higher or a 2-h plasma glucose level of 7.8 mmol/L or higher [2]. However, there is still no international agreement about the definition of gestational diabetes mellitus [3]. The prevalence of gestational diabetes mellitus is increasing worldwide [4] parallel to the increase in prevalence of overweight and obesity among pregnant women. Overweight and obesity is the strongest risk factor for gestational diabetes mellitus with 2–3 and 5–6 fold increases in the relative risk (RR) compared to normal weight women [5, 6]. Some evidence suggests an increased risk even within the high-normal range of body mass index compared to the low-normal range [7, 8], similar to what is observed for type 2 diabetes [9]. Gestational diabetes and type 2 diabetes have many pathophysiological features in common.

Although physical activity has been established as a protective factor for type 2 diabetes [10], the data regarding physical activity and gestational diabetes mellitus are less extensive and less convincing [11–37]. Several [16, 19, 21, 23–27, 29, 34–40], but not all studies [11–15, 17, 18, 20, 22, 28, 30], have reported inverse associations between higher physical activity and gestational diabetes mellitus risk, however, even among the studies that did report inverse associations the strength of the associations have varied considerably with reductions in the relative risk ranging from 10–30 [24–26, 37] up to 50–90 % [16, 19, 21, 23, 27, 34–36, 38]. It is not clear whether the variability in the results could be due to differences in the ranges and amounts of physical activity between studies, or if it varies by subtypes or intensity of physical activity, or whether it is the total amount of physical activity that is the most important factor. A previous meta-analysis of case–control and cohort studies reported an inverse association between high versus low physical activity and gestational diabetes mellitus [41], but no dose–response analyses were conducted. Two more recent meta-analyses [42, 43], which only included randomized trials came to opposite conclusions to whether physical activity reduces gestational diabetes risk with one showing no association [42] and another showing an inverse association [43], but none of the published meta-analyses reported whether the amount of physical activity was related to the outcome. Some studies have reported dose-dependent inverse associations between physical activity and gestational diabetes mellitus risk [23, 24, 31, 35, 37], however, other studies suggested that most of the benefit observed was when increasing physical activity level from a low level to a moderate level [25, 27, 34]. Clarifying whether there is a linear dose–response relationship or whether there are threshold levels of activity could be important to provide more detailed recommendations for the physical activity level needed for women to reduce the risk of gestational diabetes mellitus, and could also provide crucial information for the planning of future large-scale randomized trials of physical activity for prevention of the disease.

Several additional studies have been published since the previous meta-analyses [12–14, 16, 19–22, 28–33] and for this reason we conducted an updated systematic review and meta-analysis of physical activity and gestational diabetes mellitus with a particular aim of clarifying whether there is a dose–response relationship between increasing physical activity level and lower risk of gestational diabetes mellitus. We also summarized studies that have been published on physical activity and abnormal glucose tolerance (elevated glucose levels in the non-diabetic range) [22, 25, 33, 39, 44, 45].

## Methods

### Search strategy

The PubMed, Embase and Ovid databases were initially searched up to December 10th 2014 for cohort studies and randomized trials of physical activity and gestational diabetes mellitus risk and the searches were later updated to August 5th 2015. We used the following search terms: (physical activity OR exercise OR sports OR walking OR biking OR running OR fitness OR “exercise test” OR inactivity OR sedentary OR “risk factor” OR “risk factors”) AND (“gestational diabetes” OR “gestational diabetes mellitus”) AND (“case–control” OR retrospective OR cohort OR cohorts OR prospective OR longitudinal OR “follow-up” OR “cross-sectional” OR trial). We also searched the reference lists of previous reviews on the subject [41–43] and of the studies included in the analysis for any further studies.

### Study selection

To be included, the study had to be a randomized controlled trial, or a cohort study, and to investigate the association between physical activity and risk of gestational diabetes mellitus or abnormal glucose tolerance. Estimates of the relative risk (hazard ratio, risk ratio, odds ratio) had to be available with the 95 % confidence intervals, and for the dose–response analysis, a quantitative measure of activity level for 3 or more categories of activity and the total number of cases and person-years or participants had to be available in the publication. When multiple publications were available from the same study we used the study with the most detailed analyses of physical activity and the largest number of participants. We identified 26 studies that were included in total [11–37, 39, 44, 45], 23 studies that could be included in the analysis [11–37] of gestational diabetes mellitus and six studies that could be included in the analysis of abnormal glucose tolerance [22, 25, 33, 39, 44, 45]. A list of the excluded studies and reasons for exclusion is found in Supplementary Table 1. The search was conducted by DA and study selection was conducted by DA and AS.

### Data extraction

We extracted the following data from each study: The first author’s last name, publication year, country where the study was conducted, follow-up period, sample size, age, number of cases, exposure, physical activity level, RRs and 95 % CIs, and variables adjusted for in the analysis. Data extractions were done by DA and checked for accuracy by AS.

### Study quality assessment

The quality of the studies included was assessed using the Newcastle–Ottawa scale [46] for cohort studies and the Cochrane Collaboration’s tool for assessing risk of bias in randomised trials [47]. The Newcastle–Ottawa scale assesses the study quality based on the selection (representativeness of the exposed cohort, selection of the non-exposed cohort, ascertainment of exposure, demonstration that the outcome of interest was not present at the start of the study), comparability (adjustment for confounding factors), and the outcome (outcome assessment, long enough follow-up, adequacy of follow-up of cohorts). The randomized trials were assessed for risk of bias based on random sequence generation, allocation concealment, blinding of participants and personal, blinding of outcome assessment, incomplete outcome data, selective reporting, and other biases. Subgroup analyses by study quality scores or risk of bias were conducted separately for the observational studies and the randomized trials because of the different scales for the two study designs. For the observational studies we grouped studies with 0–3, 4–6, and 7–9 points to indicate low, medium and high quality studies, while the randomized trials studies were grouped according to whether they were at high, low or unclear risk of bias in the subgroup analyses.

### Statistical methods

We used random effects models to calculate summary RRs and 95 % CIs for the highest versus the lowest level of physical activity and for the dose–response analysis [48]. The average of the natural logarithm of the RRs was estimated and the RR from each study was weighted by the inverse of its variance and then un-weighted by a variance component which corresponds to the amount of heterogeneity in the analysis. A two-tailed *p* < 0.05 was considered statistically significant.

We used the method described by Greenland and Longnecker [49] for the dose–response analysis and computed study-specific slopes (linear trends) and 95 % CIs from the natural logs of the RRs and CIs across categories of physical activity. The method requires that the distribution of cases and person-years or non-cases and the RRs with the variance estimates for at least three quantitative exposure categories are known. We estimated the distribution of cases or person-years in studies that did not report these, but reported the total number of cases/person-years, as described previously [50, 51]. The median or mean physical activity level in each category was assigned to the corresponding relative risk for each study. For studies that reported physical activity by ranges of activity we estimated the midpoint for each category by calculating the average of the lower and upper bound. When the highest or lowest category was open-ended we assumed the open-ended interval length to be the same as the adjacent interval. For one study which only provided a continuous estimate of physical activity per 100 kcal of energy expenditure we re-calculated the odds ratio so it corresponded to an increment equal to the highest compared to the lowest quartile so it could be included in the high versus low analysis [26], and the same was done for another study [30]. For the dose–response analysis we conducted separate analyses for studies reporting results in metabolic equivalent task (MET)-hours and hours/week. The MET is an index of the intensity of physical activity and is defined as the caloric expenditure per kilogram of body weight per hour of activity, divided by the equivalent per hour at rest [52]. One MET is equal to the energy cost of a person during quiet sitting, walking slowly has a MET value of 2 and jogging and bicycling have MET values of 7–8. MET-hours are the number of hours spent in each activity multiplied with the MET value of that activity. For one study [37] we converted frequency of physical activity/week to hours/week by assigning a dose of 45 min per session [10, 53]. We examined a potential nonlinear dose–response relationship between physical activity and gestational diabetes mellitus by using restricted cubic splines with three knots at 10, 50 and 90 % percentiles of the distribution which was combined using multivariate meta-analysis [54, 55]. A likelihood ratio test was used to assess the difference between the nonlinear and linear models to test for nonlinearity [56]. Although formal dose–response analyses were not possible for the randomized controlled trials we fitted a linear regression of the RR estimates against the total number of hours/week the interventions lasted.

Heterogeneity between studies was assessed by the Q test and I^2^ [57]. I^2^ is the amount of total variation that is explained by between study variation. I^2^ values of approximately 25, 50 and 75 % are considered to indicate low, moderate and high heterogeneity, respectively. To investigate sources of heterogeneity subgroup analyses were conducted according to study design, geographic location, number of cases and adjustment for confounding factors. Meta-regression analyses were used to test for heterogeneity between subgroups.

Publication bias was assessed with Egger’s test [58] and the results were considered to indicate publication bias when *p* < 0.10. We conducted sensitivity analyses excluding one study at a time to ensure that the results were not simply due to one large study or a study with an extreme result. The statistical analyses were conducted using Stata, version 13.0 software (StataCorp, College Station, TX, USA). The following Stata commands were used: metan7 (high versus low analyses and dose–response analyses), glst (dose–response analyses), metareg (meta-regression analyses), metabias6 (Egger’s test), metafunnel (funnel plot), metaninf (influence or sensitivity analyses).

## Results

**Fig. 1:**
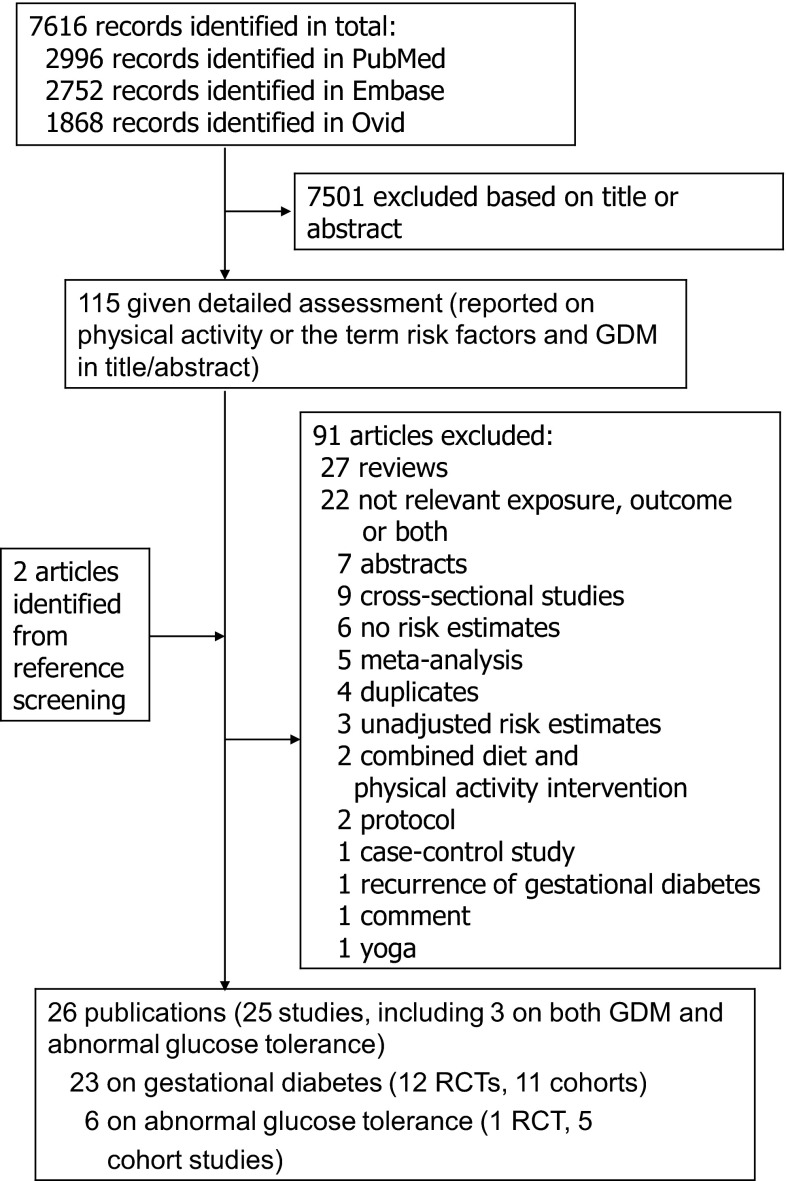
Flow-chart of study selection

Out of the 7616 records which were identified by the literature search, 7501 were excluded based on the title and abstract (one author screened all references), and 115 full text articles were assessed in detail (in duplicate) as they reported on physical activity or risk factors and gestational diabetes mellitus in the title/abstract (Fig. 1). Out of these articles, 26 publications (25 studies) were finally included in the meta-analysis, including twelve randomized trials [11–22] and eleven cohort studies [23–33] that could be included in the meta-analysis of physical activity and gestational diabetes mellitus (Tables 1, 2, 3; Fig. 1), and one randomized trial [22] and five cohort studies [25, 33, 39, 44, 45] that were included in the meta-analysis of physical activity and risk of abnormal glucose tolerance (Table 4; Fig. 1).

**Table 1:** Randomized controlled trials of physical activity and gestational diabetes mellitus risk

Author, publication year, country/region	Follow-up period	Study size, gender, age, number of cases	Exposure	Quantity	RR (95 % CI)^a^	Adjustment for confounders
Callaway LK et al., 2010, Australia	NA	22 obese women, exercise intervention: 5 cases	*Physical activity during pregnancy*	900 kcal/week of energy expenditure	1.44 (0.43–4.98)	–
		19 controls: 3 cases	Individualised exercise program with the goal of an energy expenditure of 900 kcal/week			
Price BB et al., 2012, USA	2006–2010	43 (31 analysed) women, exercise intervention: 3 cases	*Physical activity during pregnancy*	45–60 min × 4/week	0.75 (0.20–2.78)	–
		48 (31 analysed) women, control group: 4 cases	Supervised aerobic training of 45–60 min 4 times per week at moderate intensity (12–14 on Borg Scale of perceived exertion)			
Oostdam N et al., 2012, Netherlands	2007–2011	62 (48) women, exercise group: 7 cases	*Physical activity during pregnancy*	60 min × 2/week	0.65 (0.27–1.55)	–
		59 (51) women, control group: 11 cases	Exercise program 2 days/week, 60 min per session. Aerobic and strength exercises aimed to control blood glucose levels			
Stafne SN et al., 2012, Norway	2007–2009	429 (375 analysed) women, exercise group: 25 cases	*Physical activity during pregnancy*	60 min × 1/week	1.21 (0.68–2.16)	–
		426 (327 analysed) women, control group: 18 cases	Standardized exercise program including aerobic activity, strength training, balance exercises, 60 min training sessions one time per week between gestational week 20–36			
Barakat R et al., 2012, Spain	NA	40 women, exercise group: 0 cases	*Physical activity during pregnancy*	35–45 min × 3/week	0.15 (0.01–1.32)	–
		43 women, control group: 3 cases	Physical conditioning programme with two land aerobic sessions and one aquatic session of 35–45 min sessions for a total of 3 times per week from gestational weeks 6–9 to weeks 38–39			
Barakat R et al., 2013, Spain	2007–2011	255 women, exercise group: 41 cases	*Physical activity during pregnancy*	50–55 min × 3/week	0.84 (0.50–1.40)	Maternal age, pre-pregnancy body weight
		255 women, control group: 61 cases	Physical activity intervention 50–55 min per session, 3 times per week including aerobic exercise, muscle strengthening and flexibility exercises from weeks 10–12 of pregnancy to weeks 38–39			
Tomic V et al., 2013, Croatia	2008–2009	166 women, physical exercise group: 3 cases	*Physical activity during pregnancy*	50 min × 3/week	0.22 (0.07–0.69)	–
		168 women, control group: 14 cases	Aerobic exercise of moderate intensity, 50 min/session, 3 times/week during the whole pregnancy period			
Barakat R et al., 2014, Spain	NA	137 women, exercise group: 5 cases	*Physical activity during pregnancy*	55–60 min × 3/week	0.83 (0.26–2.63)	–
		114 women, control group: 5 cases	Physical conditioning program of three 55–60 min sessions per week that started between 9 and 13 weeks gestation to weeks 39–40			
Ko CW et al., 2014, USA	NA	591 women, physical activity group: 25 cases	*Physical activity during pregnancy*	30 min × 3/week − 45–60 min/week × 4–5/week	0.78 (0.47–1.28)	–
		605 women, control group: 33 cases	Exercise intervention of moderate to vigorous intensity, 30 min/session, 3 times per week with a goal to increase to 45–60 min/session, 4–5 times/week			
Renault KM et al., 2014, Denmark	2009–2012	125 women, physical activity group: 2 cases	*Physical activity during pregnancy*	11,000 steps/day	0.27 (0.06–1.16)	–
		134 women, control group: 7 cases	Physical activity intervention to increase daily step count to 11,000 (monitored by pedometer)			
Cordero Y et al., 2014, Spain	NA	101 women, exercise group: 1 case	*Physical activity during pregnancy*	50–60 min × 3/week	0.10 (0.01–0.80)	–
		156 women, control group: 13 cases	Physical activity program with 50–60 min sessions, 3 times per week from weeks 10–14 to the end of the third trimester			
Nobles C et al., 2015, USA	2007–2012	124 women, exercise group: 12 cases	*Physical activity during pregnancy*	30 min most days of the week	0.60 (0.27–1.32)	Education, parity
		127 women, control group: 19 cases	Intervention to increase activity level to moderate-intensity physical activity of 30 min on most days of the week			

*NA* not available

^a^All relative risk estimates were estimated based on the distribution of cases and participants apart from the study by Barakat et al. [17] and Cordero et al. [21]

**Table 2:** Prospective cohort studies of physical activity and gestational diabetes mellitus risk

Author, publication year, country/region	Follow-up period	Study size, gender, age, number of cases	Exposure	Quantity	RR (95 % CI)	Adjustment for confounders
Dempsey JC et al., 2004, USA	1996–2000	909: 42 cases	*Physical activity before pregnancy*			
			Any recreational physical activity	No	1.00	Age, race, parity
				Yes	0.36 (0.18–0.76)	
			Time spent performing recreational physical activity	None	1.00	
				<4.2 hrs/wk	0.51 (0.24–1.09)	
				≥4.2	0.20 (0.08–0.51)	
			Energy expended performing recreational physical activity	None	1.00	
				<21.1 MET-hours/week	0.51 (0.24–1.08)	
				≥21.1	0.20 (0.08–0.51)	
			Any recreational physical activity	No	1.00	Age, race, parity, prepregnancy BMI
				Yes	0.44 (0.21–0.91)	
			Time spent performing recreational physical activity	None	1.00	
				<4.2 hrs/wk	0.58 (0.27–1.24)	
				≥4.2	0.24 (0.10–0.64)	
			Energy expended performing recreational physical activity	None	1.00	
				<21.1 MET-hours/week	0.57 (0.27–1.21)	
				≥21.1	0.26 (0.10–0.65)	
			*Physical activity during pregnancy*			
			Any recreational physical activity	No	1.00	Age, race, parity
				Yes	0.60 (0.33–1.10)	
			Time spent performing recreational physical activity	None	1.00	
				<6.0 hrs/wk	0.43 (0.19–0.99)	
				≥6.0	0.76 (0.38–1.50)	
			Energy expended performing recreational physical activity	None	1.00	
				<28 MET-hours/week	0.65 (0.32–1.34)	
				≥28	0.55 (0.26–1.17)	
			Any recreational physical activity	No	1.00	Age, race, parity, prepregnancy BMI
				Yes	0.69 (0.37–1.29)	
			Time spent performing recreational physical activity	None	1.00	
				<6.0 hrs/wk	0.49 (0.21–1.13)	
				≥6.0	0.90 (0.45–1.80)	
			Energy expended performing recreational physical activity	None	1.00	
				<28 MET-hours/week	0.71 (0.35–1.47)	
				≥28	0.67 (0.31–1.43)	
			Recreational physical activity before and during pregnancy	Neither	1.00	Age, race, parity
				Active last year only	0.45 (0.17–1.20)	
				Active last week only	0.83 (0.23–2.94)	
				Active both periods	0.28 (0.11–0.73)	
			Recreational physical activity before and during pregnancy	Neither	1.00	Age, race, parity, prepregnancy BMI
				Active last year only	0.40 (0.15–1.07)	
				Active last week only	0.59 (0.16–2.14)	
				Active both periods	0.31 (0.12–0.79)	
Oken E et al., 2006, USA	1999–2002	1805: 91 cases	*Physical activity before pregnancy*			
			Walking	≤1 hrs/wk	1.00	Age, race/ethnicity, prepregnancy BMI, history of gestational diabetes mellitus in a previous pregnancy, mother’s history of gestational diabetes mellitus
				>1	0.67 (0.34–1.34)	
			Light moderate activity	None	1.00	
				Any	1.16 (0.67–1.99)	
			Vigorous activity	None	1.00	
				Any	0.56 (0.33–0.95)	
			Light/moderate or vigorous activity	None	1.00	
				Any	0.91 (0.51–1.65)	
			Total leisure-time physical activity	≤2 hrs/wk	1.00	
				3–6	0.78 (0.34–1.76)	
				7–13	0.63 (0.28–1.39)	
				≥14	0.70 (0.30–1.68)	
			*Physical activity during pregnancy*			
			Walking, during pregnancy	≤1 hrs/wk	1.00	
				>1	0.67 (0.35–1.30)	
			Light moderate activity	None	1.00	
				Any	0.70 (0.41–1.21)	
			Vigorous activity	None	1.00	
				Any	0.90 (0.47–1.70)	
			Light/moderate or vigorous activity	None	1.00	
				Any	0.73 (0.43–1.25)	
			Total leisure-time physical activity	≤2 hrs/wk	1.00	
				3–6	0.72 (0.37–1.40)	
				7–13	0.59 (0.28–1.23)	
				≥14	0.91 (0.37–2.21)	
			*Physical activity before and during pregnancy*	No/no	1.00	
				No/yes	1.28 (0.54–3.02)	
				Yes/no	0.83 (0.36–1.90)	
				Yes/yes	0.49 (0.24–1.01)	
Zhang C et al., 2006, USA	1989–1999, 10 years follow-up	21,765: 1428 cases	*Physical activity before pregnancy*			
			Leisure-time physical activity, prepregnancy	2.3 MET-hours/week	1.00	Age, race/ethnicity, smoking status, family history of diabetes mellitus in 1st degree relative, parity, alcohol intake, total energy, cereal fiber, total fat, glycemic load
				7.8	0.94 (0.83–1.14)	
				15.9	0.83 (0.70–0.97)	
				29	0.86 (0.73–1.02)	
				63.2	0.71 (0.60–0.84)	
			Vigorous physical activity	0 MET-hours/week	1.00	
				1.4	0.94 (0.71–1.26)	
				6	0.81 (0.67–1.16)	
				15	0.70 (0.60–0.82)	
				38.8	0.72 (0.62–0.84)	
			Usual walking pace, among women without vigorous activity	Casual	1.00	
				Normal	0.79 (0.57–1.08)	
				Brisk, very brisk	0.60 (0.42–0.86)	
			Stair climbing, among women without vigorous activity	≤2 flights/day	1.00	
				3–4	1.03 (0.80–1.33)	
				5–9	0.97 (0.75–1.26)	
				10–14	0.73 (0.51–1.05)	
				≥15	0.44 (0.24–0.79)	
			Leisure-time physical activity	2.3 MET-hours/week	1.00	Age, race/ethnicity, smoking status, family history of diabetes mellitus in 1st degree relative, parity, alcohol intake, total energy, cereal fiber, total fat, glycemic load, BMI
				7.8	0.97 (0.87–1.20)	
				15.9	0.88 (0.75–1.04)	
				29	0.90 (0.80–1.11)	
				63.2	0.81 (0.68–1.01)	
			Vigorous physical activity	0 MET-hours/week	1.00	
				1.4	0.95 (0.71–1.28)	
				6	0.84 (0.71–1.20)	
				15	0.75 (0.64–0.87)	
				38.8	0.77 (0.69–0.94)	
			Usual walking pace, among women without vigorous activity	Casual	1.00	
				Normal	0.81 (0.59–1.12)	
				Brisk, very brisk	0.66 (0.46–0.95)	
			Stair climbing, among women without vigorous activity	≤2 flights/day	1.00	
				3–4	1.03 (0.80–1.33)	
				5–9	1.00 (0.77–1.30)	
				10–14	0.77 (0.54–1.11)	
				≥15	0.50 (0.27–0.90)	
Iqbal R et al., 2007, Pakistan	2002–2004	611 South Asian women: 49 cases	*Physical activity before pregnancy*			
			Total physical activity	Per 100 kcal energy expenditure	0.89 (0.79–0.99)	Age, BMI, family history of diabetes, education, parity, height, rate of weight gain during pregnancy
			Total physical activity	Per 100 kcal energy expenditure	0.89 (0.79–0.99)	Age, body fat, family history of diabetes, education, parity, height, rate of weight gain during pregnancy
Chasan-Taber L et al., 2008, USA	2000–2004	1006 Hispanic women: 33 cases	*Physical activity before pregnancy*			
			Household activity, caregiving	1.9 KPAS score	1.0	Age, BMI
				2.3	0.3 (0.1–0.9)	
				2.7	0.3 (0.1–0.9)	
				3.2	0.2 (0.1–0.8)	
			Occupational physical activity	1.0 KPAS score	1.0	
				2	0.4 (0.1–2.2)	
				3	2.1 (0.7–6.0)	
				3.9	1.7 (0.6–5.1)	
			Sports, exercise	1.3 KPAS score	1.0	
				1.5	1.8 (0.5–6.0)	
				2.8	1.5 (0.4–5.8)	
				4	2.1 (0.6–7.1)	
			Active living	1.7 KPAS score	1.0	
				2.5	1.1 (0.3–3.7)	
				3	1.3 (0.4–3.8)	
				3.8	2.0 (0.6–6.2)	
			Total physical activity	7.9 KPAS score	1.0	
				9.5	0.5 (0.1–2.3)	
				10.8	2.0 (0.7–5.8)	
				12.3	0.8 (0.2–2.7)	
			*Physical activity during early pregnancy*			
			Household activity, caregiving	1.7 KPAS score	1.0	
				2.1	0.3 (0.1–1.3)	
				2.4	0.7 (0.2–1.9)	
				3	0.8 (0.3–2.3)	
			Occupational physical activity	1.0 KPAS score	1.0	
				2.3	0.6 (0.2–1.9)	
				3.3	1.5 (0.7–3.5)	
			Sports, exercise	1.0 KPAS score	1.0	
				1.3	1.5 (0.5–4.5)	
				1.5	0.9 (0.3–2.9)	
				2.3	0.7 (0.2–2.5)	
			Active living	1.3 KPAS score	1.0	
				2	0.5 (0.1–1.7)	
				2.7	1.5 (0.5–4.6)	
				3.3	1.2 (0.4–3.4)	
			Total physical activity	6.8 KPAS score	1.0	
				8.1	0.5 (0.1–1.6)	
				9.2	0.6 (0.2–1.7)	
				10.7	0.8 (0.2–2.3)	
			*Physical activity during midpregnancy*			
			Household activity, caregiving	1.6 KPAS score	1.0	
				2	0.8 (0.3–2.4)	
				2.4	0.2 (0.1–0.7)	
				2.9	0.2 (0.1–0.8)	
			Occupational physical activity	1.0 KPAS score	1.0	
				2.2	0.7 (0.2–3.0)	
				3.1	1.0 (0.4–2.6)	
			Sports, exercise	1.0 KPAS score	1.0	
				1.3	0.4 (0.1–1.4)	
				1.5	1.3 (0.5–3.7)	
				2.5	0.1 (0.0–0.7)	
			Active living	1.5 KPAS score	1.0	
				2	0.6 (0.1–2.6)	
				2.7	1.9 (0.7–5.6)	
				3.3	0.6 (0.2–2.1)	
			Total physical activity	6.7 KPAS score	1.0	
				8.1	0.6 (0.2–1.8)	
				9.2	0.3 (0.1–1.1)	
				10.6	0.4 (0.1–1.2)	
Van der Ploeg HP et al., 2011, Australia	1978–2003	2913: 180 cases	*Physical activity before pregnancy*			
			Moderate and vigorous physical activity	<180 MET min/wk	1.00	Age, number of children, age at 1st birth, country of birth, education, total energy intake
				180–600	1.28 (0.80–2.06)	
				600–1320	1.53 (0.95–2.47)	
				>1320	1.22 (0.70–2.11)	
Ramos-Levi AM et al., 2012, Spain	2009–2010	2194: 213 cases	*Physical activity before pregnancy*			
			Sports	≥2 versus <2 days/wk	0.67 (0.45–0.96)	Age, biscuits and pastries, red and processed meats, fruit, dried fruits and nuts, skimmed dairy products, legumes, blue fish, whole wheat bread, sauces, vegetables and salads, water, alcohol, sugary drinks, coffee, light walking, climbing up stairs
Mørkrid K et al., 2014, Norway	2008–2010	759 women: 239 cases	*Physical activity before pregnancy*			
			Regular physical activity before pregnancy	No	1.00	Age, gestational week, pre-pregnancy BMI, ethnic origin, early life socioeconomic position score
				Yes	0.65 (0.46–0.94)	
			*Physical activity during early pregnancy*			
			Objectively recorded moderate-vigorous physical activity	Per 1 SD (60 min/d)	1.02 (0.85–1.23)	
			Objectively recorded steps	Per 1 SD (3159 steps/d)	0.81 (0.66–0.99)	
Zhang C et al., 2014, USA	1989–2001	14,437 women: 823 cases	*Physical activity before pregnancy*			
			Moderate/vigorous physical activity	<30 min/wk	1.00	Age, parity, family history of diabetes, history of infertility, race/ethnicity, questionnaire period, total energy, alcohol, Alternate Healthy Eating Index-2010 diet score, BMI, smoking status
				30–59	0.90 (0.72–1.13)	
				60–89	0.91 (0.71–1.16)	
				90–149	0.89 (0.71–1.10)	
				150–209	0.85 (0.66–1.10)	
				≥210	0.78 (0.64–0.94)	
Currie LM et al., 2014, Canada	2002–2005	1749 women: 36 cases	*Physical activity before pregnancy*			
			Total physical activity	<7.72 KPAS score	1.00	Maternal age, prepregnancy BMI, education, parity, history of gestational diabetes
				7.72- < 9.39	0.71 (0.33–1.56)	
				≥9.39	0.60 (0.24–1.48)	
			*Physical activity in the 1st half of pregnancy*			
			Total physical activity	<6.44 KPAS score	1.00	
				6.44–<7.97	1.08 (0.50–2.32)	
				≥7.97	0.56 (0.22–1.47)	
Chasan-Taber L et al., 2014, USA	2006–2011	1241 women: 175 cases	*Physical activity before pregnancy*			
			Met exercise guidelines, prepregnancy	No	1.00	Age, pre-pregnancy BMI, education, generation in the US
				Yes	1.01 (0.55–1.83)	
			Total physical activity	1	1.00	
				2	1.20 (0.52–2.76)	
				3	1.13 (0.50–2.57)	
				4	0.79 (0.32–1.97)	
			Moderate-intensity physical activity	1	1.00	
				2	0.97 (0.44–2.14)	
				3	1.07 (0.49–2.35)	
				4	0.69 (0.29–1.66)	
			Vigorous intensity physical activity	No	1.00	
				Yes	0.90 (0.48–1.69)	
			Household/caregiving physical activity	1	1.00	
				2	1.25 (0.58–2.69)	
				3	0.73 (0.31–1.71)	
				4	0.61 (0.26–1.45)	
			Occupational physical activity	1	1.00	
				2	2.98 (1.26–7.03)	
				3	1.38 (0.51–3.76)	
				4	2.05 (0.86–4.90)	
			Sports/exercise	1	1.00	
				2	1.76 (0.78–3.95)	
				3	0.96 (0.39–2.36)	
				4	1.26 (0.52–3.05)	
			*Physical activity during early pregnancy*			
			Met exercise guidelines, early pregnancy	No	1.00	
				Yes	0.91 (0.45–1.83)	
			Total physical activity	1	1.00	
				2	0.43 (0.14–1.27)	
				3	0.92 (0.39–2.18)	
				4	0.69 (0.27–1.73)	
			Moderate-intensity physical activity	1	1.00	
				2	1.12 (0.47–2.65)	
				3	0.76 (0.30–1.94)	
				4	0.64 (0.24–1.76)	
			Vigorous intensity physical activity	No	1.00	
				Yes	1.07 (0.39–2.89)	
			Household/caregiving physical activity	1	1.00	
				2	2.75 (0.99–7.58)	
				3	1.08 (0.34–3.41)	
				4	1.88 (0.66–5.36)	
			Occupational physical activity	Unemployed	1.00	
				Low	0.82 (0.38–1.77)	
				High	0.39 (0.15–0.99)	
			Sports/exercise	1	1.00	
				2	1.51 (0.31–7.30)	
				3	1.11 (0.49–2.51)	
				4	1.07 (0.47–2.44)	

*Hrs* hours, *wk* week, *KPAS* Kaiser Physical Activity Survey, *MET* metabolic equivalent task, *min* minutes

**Table 3:** Cohort studies and randomized trials of physical activity and impaired or abnormal glucose tolerance

Author, publication year, country/region	Study period	Study size, gender, age, number of cases	Exposure	Quantity	RR (95 % CI)	Adjustment for confounders
Oken E et al., 2006, USA	1999–2002	1805 women: 312 abnormal glucose tolerance cases	*Physical activity before pregnancy*			
			Walking	≤1 h/week	1.00	Age, race/ethnicity, prepregnancy BMI, history of gestational diabetes mellitus in a previous pregnancy, mother’s history of gestational diabetes mellitus
				>1	0.77 (0.52–1.13)	
			Light moderate activity	None	1.00	
				Any	1.15 (0.86–1.53)	
			Vigorous activity	None	1.00	
				Any	0.76 (0.57–1.00)	
			Light/moderate or vigorous activity	None	1.00	
				Any	1.00 (0.73–1.37)	
			Total leisure-time physical activity	≤2 h/week	1.00	
				3–6	0.84 (0.54–1.31)	
				7–13	0.82 (0.53–1.25)	
				≥14	0.79 (0.49–1.26)	
			*Physical activity during pregnancy*			
			Walking, during pregnancy	≤1 h/week	1.00	
				>1	0.71 (0.49–1.02)	
			Light moderate activity	None	1.00	
				Any	0.79 (0.60–1.05)	
			Vigorous activity	None	1.00	
				Any	0.73 (0.52–1.03)	
			Light/moderate or vigorous activity	None	1.00	
				Any	0.76 (0.57–1.00)	
			Total leisure-time physical activity	≤2 h/week	1.00	
				3–6	0.94 (0.65–1.34)	
				7–13	0.80 (0.54–1.19)	
				≥14	0.68 (0.40–1.16)	
			*Physical activity before and during pregnancy*	No/no	1.00	
				No/yes	1.19 (0.74–1.91)	
				Yes/no	1.04 (0.67–1.60)	
				Yes/yes	0.70 (0.49–1.01)	
Gollenberg AL et al., 2010, USA	2000–2003 2006–2008	1231 Latina women: 104 abnormal glucose tolerance cases	*Physical activity before pregnancy*			
			Sports, exercise	1	1.00 (0.51–1.96)	Maternal age, smoking, pre-pregnancy BMI, maternal education, parity
				2	1.67 (0.94–2.97)	
				3	1.04 (0.54–2.03)	
				4	1.00	
			*Physical activity during early pregnancy*			
			Sports, exercise	1	1.16 (0.60–2.24)	
				2	1.66 (0.92–2.98)	
				3	1.30 (0.69–2.45)	
				4	1.00	
Baptiste-Roberts K et al., 2011, USA		152 women: not available	*Physical activity before pregnancy*			
			Leisure-time physical activity	≥2.75 versus <2.75 score	0.32 (0.12–0.86)	Age, race, parity, gestational weight gain, pre-pregnancy BMI
Deierlein AL et al., 2012, USA	2001–2005	1437 women: 269 hyperglycemia cases	*Physical activity during early pregnancy*			
			Total moderate and vigorous physical activity	None	1.00	Age at conception, pre-pregnancy BMI, race/ethnicity
				Any	0.93 (0.74–1.17)	
			Recreational moderate and vigorous physical activity	None	1.00	
				Any	0.73 (0.54–0.99)	
			Outdoor household moderate and vigorous physical activity	None	1.00	
				Any	0.90 (0.49–1.66)	
			Indoor household moderate and vigorous physical activity	None	1.00	
				Any	1.10 (0.76–1.60)	
			Caregiving moderate and vigorous physical activity	None	1.00	
				Any	0.80 (0.43–1.51)	
			Work-related moderate and vigorous physical activity	None	1.00	
				Any	1.26 (0.90–1.78)	
Chasan-Taber L et al., 2014, USA	2006–2011	1241 women: 161 abnormal glucose tolerance cases	*Physical activity before pregnancy*			
			Met exercise guidelines, prepregnancy	No	1.00	Age, pre-pregnancy BMI, education, generation in the US
				Yes	1.09 (0.76–1.56)	
			Total physical activity	1	1.00	
				2	0.67 (0.40–1.13)	
				3	0.81 (0.49–1.33)	
				4	0.94 (0.57–1.53)	
			Moderate-intensity physical activity	1	1.00	
				2	1.07 (0.67–1.72)	
				3	0.72 (0.43–1.19)	
				4	0.91 (0.56–1.50)	
			Vigorous intensity physical activity	No	1.00	
				Yes	1.05 (0.73–1.51)	
			Household/caregiving physical activity	1	1.00	
				2	0.88 (0.54–1.44)	
				3	0.91 (0.56–1.49)	
				4	0.89 (0.55–1.45)	
			Occupational physical activity	1	1.00	
				2	1.12 (0.68–1.82)	
				3	0.97 (0.58–1.64)	
				4	0.97 (0.58–1.64)	
			Sports/exercise	1	1.00	
				2	1.18 (0.72–1.92)	
				3	0.98 (0.59–1.62)	
				4	1.01 (0.61–1.68)	
			*Physical activity in early pregnancy*			
			Met exercise guidelines, early pregnancy	No	1.00	
				Yes	1.12 (0.73–1.73)	
			Total physical activity	1	1.00	
				2	0.70 (0.40–1.24)	
				3	0.60 (0.34–1.07)	
				4	0.63 (0.36–1.12)	
			Moderate-intensity physical activity	1	1.00	
				2	0.61 (0.35–1.05)	
				3	0.65 (0.38–1.12)	
				4	0.49 (0.27–0.89)	
			Vigorous intensity physical activity	No	1.00	
				Yes	0.70 (0.35–1.39)	
			Household/caregiving physical activity	1	1.00	
				2	0.65 (0.37–1.14)	
				3	0.58 (0.32–1.03)	
				4	0.67 (0.38–1.18)	
			Occupational physical activity	Unemployed	1.00	
				Low	0.92 (0.57–1.47)	
				High	0.47 (0.27–0.82)	
			Sports/exercise	1	1	
				2	1.70 (0.64–4.52)	
				3	1.25 (0.77–2.03)	
				4	1.04 (0.62–1.74)	
Nobles C et al., 2015, USA	2007–2012	124 women, exercise group: 17 cases of impaired glucose tolerance	*Physical activity during pregnancy*			
		127 women, control group: 24 cases	Intervention to increase activity level to moderate-intensity physical activity of 30 min on most days of the week	30 min most days of the week	0.68 (0.35–1.34)	Education, parity

**Table 4:** Subgroup analyses of leisure-time physical activity and gestational diabetes mellitus risk, high versus low analysis

	Leisure-time physical activity before pregnancy					Leisure-time physical activity during pregnancy				
	*n*	RR (95 % CI)	*I* ^2^ (%)	*P*_h_^a^	*P*_h_^b^	*n*	RR (95 % CI)	*I* ^2^ (%)	*P*_h_^a^	*P*_h_^b^
All studies	8	0.78 (0.61–1.00)	47.1	0.07		17	0.80 (0.64–1.00)	17.0	0.26	
Study design										
Randomized controlled trials	0				0.36	12	0.69 (0.50–0.96)	30.2	0.15	0.61
Cohort studies	8	0.78 (0.61–1.00)	47.1	0.07		5	0.97 (0.73–1.28)	0	0.80	
Geographic location										
Europe	2	0.66 (0.51–0.86)	0	0.91	0.41	9	0.67 (0.44–1.01)	52.0	0.03	0.88
America	5	0.79 (0.48–1.29)	55.6	0.06		7	0.79 (0.58–1.08)	0	0.94	
Asia	0					0				
Australia	1	1.22 (0.70–2.11)				1	1.44 (0.43–4.98)			
Number of cases										
Cases <200	5	0.88 (0.47–1.65)	62.2	0.03	0.48	16	0.75 (0.59–0.96)	13.2	0.30	0.24
Cases ≥200	3	0.75 (0.64–0.88)	0	0.46		1	1.04 (0.73–1.49)			
Study quality (observational studies)										
0–3	0				0.45	0				0.71
4–6	4	0.91 (0.59–1.42)	44.6	0.14		2	0.83 (0.40–1.73)	0	0.74	
7–9	4	0.70 (0.48–1.01)	59.6	0.06		3	0.99 (0.73–1.34)	0	0.51	
Risk of bias (randomized trials)										
High risk of bias	–				–	6	0.62 (0.31–1.24)	57.9	0.04	0.98
Low risk of bias	–					1	0.65 (0.27–1.55)			
Unclear risk of bias	–					5	0.74 (0.53–1.03)	0	0.44	
*Adjustment for confounding factors* ^*c*^										
Age										
Yes	8	0.78 (0.61–1.00)	47.1	0.07	NC	7	0.84 (0.62–1.13)	22.4	0.26	NC
No	0					0				
Education										
Yes	2	1.23 (0.77–1.97)	0	0.95	0.14	4	0.50 (0.21–1.24)	56.5	0.08	0.31
No	6	0.70 (0.54–0.91)	45.2	0.10		4	0.94 (0.70–1.26)	0	0.74	
Income										
Yes	0				NC	0				NC
No	8	0.78 (0.61–1.00)	47.1	0.07		5	0.96 (0.71–1.29)	0	0.80	
Parity										
Yes	2	0.51 (0.17–1.51)	81.6	0.02	0.33	3	0.59 (0.33–1.05)	0	0.52	0.17
No	6	0.84 (0.62–1.13)	35.9	0.17		4	1.02 (0.76–1.38)	0	0.89	
Alcohol										
Yes	2	0.78 (0.65–0.93)	0	0.38	0.82	0				NC
No	6	0.82 (0.51–1.30)	59.9	0.03		5	0.97 (0.73–1.28)	0	0.80	
Smoking										
Yes	1	0.81 (0.68–1.01)			0.93	0				NC
No	7	0.78 (0.55–1.10)	53.4	0.05		5	0.97 (0.73–1.28)	0	0.80	
Body mass index										
Yes	6	0.75 (0.54–1.04)	50.3	0.07	0.71	7	0.84 (0.62–1.13)	22.4	0.26	NC
No	2	0.87 (0.49–1.56)	67.5	0.08		0				

*n* denotes the number of studies, *NC* not calculable

^a^P for heterogeneity within each subgroup

^b^P for heterogeneity between subgroups with meta-regression analysis

^c^ *n* may not add up to the total because most of the randomized trials did not adjust for confounding factors

### Total physical activity before pregnancy

**Fig. 2:**
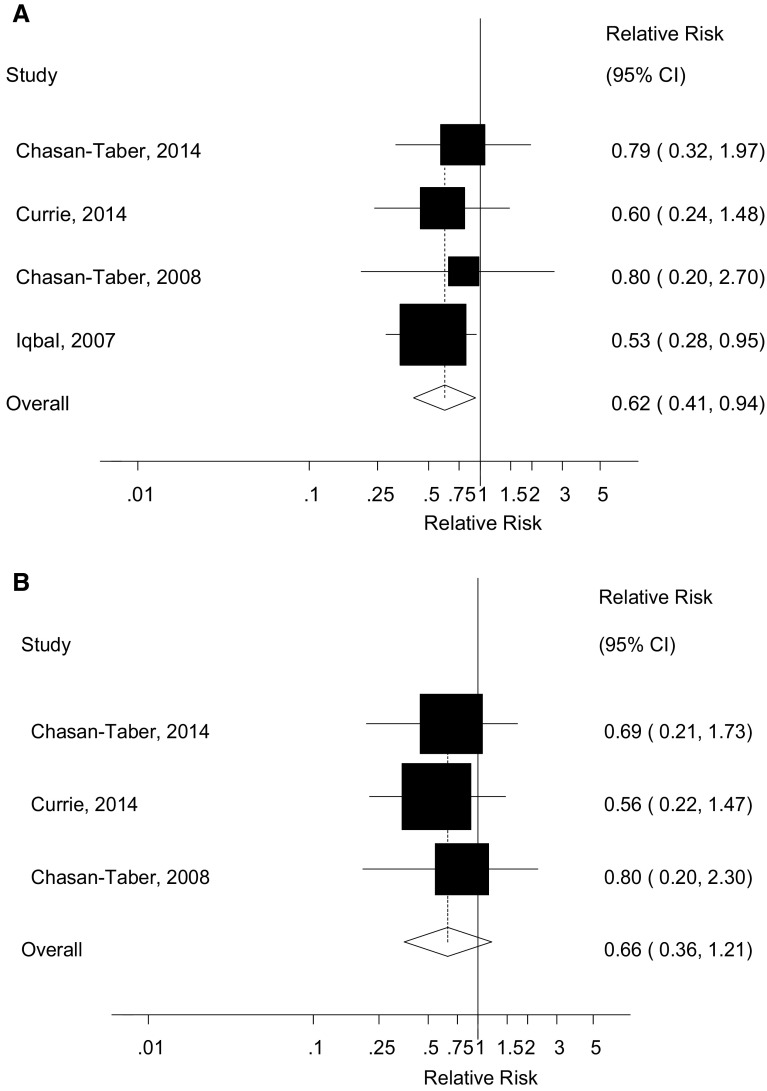
Total physical activity before and during pregnancy and gestational diabetes, high versus low comparison. **a** Total physical activity before pregnancy and gestational diabetes mellitus, high versus low analysis. **b** Total physical activity during pregnancy and gestational diabetes mellitus, high versus low analysis

Four cohort studies [26, 27, 32, 33] were included in the analysis of total physical activity (sum of leisure-time, household and occupational activity) before pregnancy and gestational diabetes mellitus and included 293 cases and 4607 participants. The summary RR for high versus low activity was 0.62 (95 % CI 0.41–0.94, I^2^ = 0 %, p_heterogeneity_ = 0.88) (Fig. 2a). Because of differences in the way the results were reported it was not possible to conduct dose–response analyses of total physical activity.

### Total physical activity during pregnancy

Three cohort studies [27, 32, 33] were included in the analysis of total physical activity during pregnancy and gestational diabetes mellitus and included 244 cases and 3996 participants. The summary RR for high versus low activity was 0.66 (95 % CI 0.36–1.21, I^2^ = 0 %, p_heterogeneity_ = 0.90) (Fig. 2b). Because of differences in the way the results were reported it was not possible to conduct dose–response analyses of total physical activity.

### Leisure-time physical activity before pregnancy

**Fig. 3:**
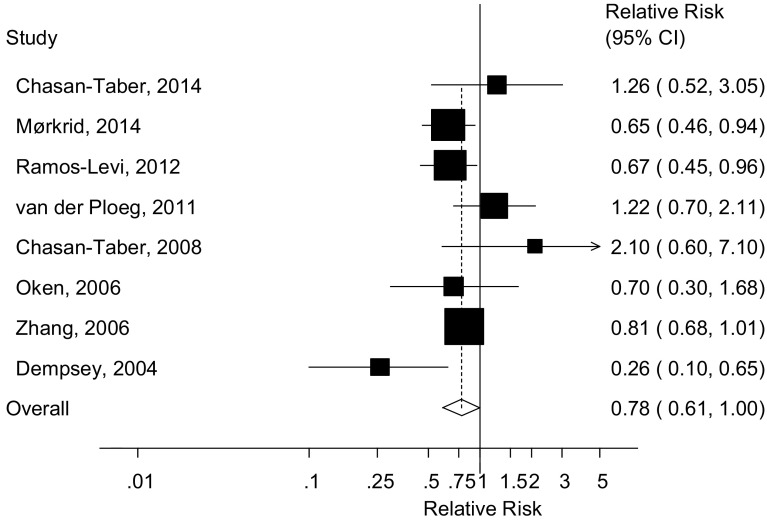
Leisure-time physical activity before pregnancy and gestational diabetes mellitus, highest versus lowest comparison

**Fig. 4:**
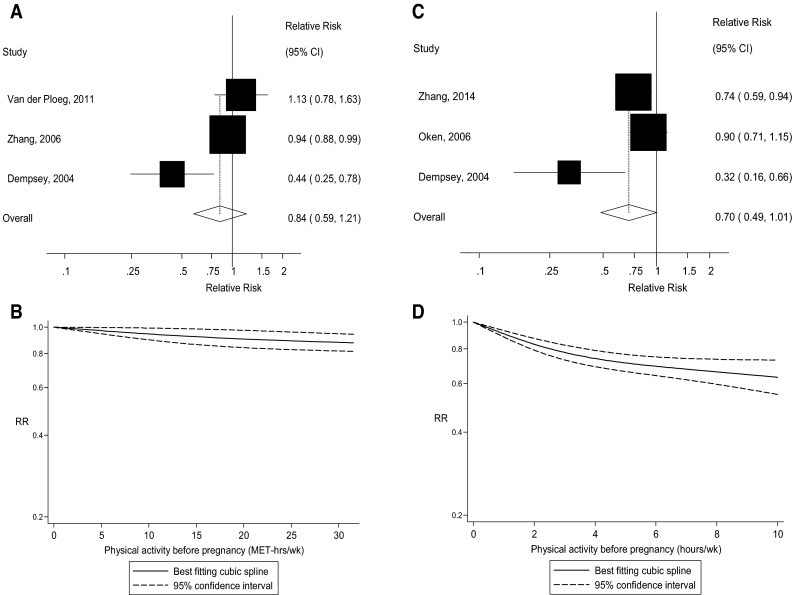
Leisure-time physical activity before pregnancy and gestational diabetes mellitus, linear (per 20 MET-hours/week and per 5 h/week) and nonlinear dose–response analysis. **a** Leisure-time physical activity before pregnancy and gestational diabetes mellitus, per 20 MET-hours/week. **b** Leisure-time physical activity before pregnancy and gestational diabetes mellitus, nonlinear dose–response, MET-hours/week. **c** Leisure-time physical activity before pregnancy and gestational diabetes mellitus, per 5 h/week. **d** Leisure-time physical activity before pregnancy and gestational diabetes mellitus, nonlinear dose–response, h/week

Eight cohort studies [23–25, 27–30, 33] were included in the analysis of pre-pregnancy physical activity and the risk of gestational diabetes mellitus and included 2401 cases and 32,592 participants. The summary RR for high versus low pre-pregnancy physical activity was 0.78 (95 % CI 0.61–1.00, I^2^ = 47 %, p_heterogeneity_ = 0.07) (Fig. 3). There was no evidence of publication bias with Egger’s test, *p* = 0.87. In the dose–response analysis of MET-hours/week the summary RR was 0.84 (95 % CI 0.59–1.21, I^2^ = 80.9 %, p_heterogeneity_ = 0.001) per 20 MET-hours/week [23, 24, 28] (Fig. 4a) and there was no evidence of nonlinearity, *p* = 0.31 (Fig. 4b; Supplementary Table 2). In the dose–response analysis of hours/week the summary RR was 0.70 (95 % CI 0.49–1.01, I^2^ = 72.6 %, p_heterogeneity_ = 0.03) per 5 h/week [23, 25, 31] (Fig. 4c). There was evidence of nonlinearity, p_nonlinearity_ = 0.005, with a steeper inverse association at the lower levels of physical activity, but further reductions in risk were observed with higher levels of activity (Fig. 4d; Supplementary Table 2).

### Leisure-time physical activity during pregnancy

**Fig. 5:**
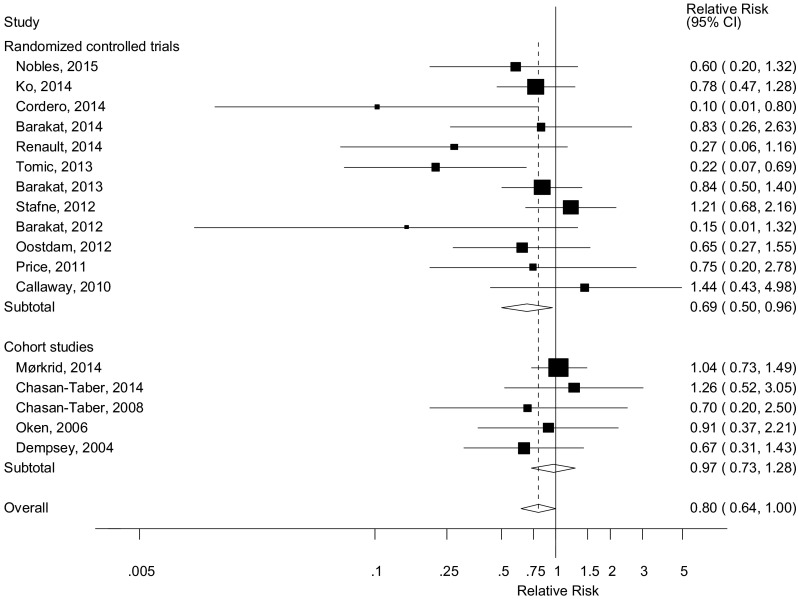
Leisure-time physical activity during early pregnancy and gestational diabetes mellitus, high versus low comparison

**Fig. 6:**
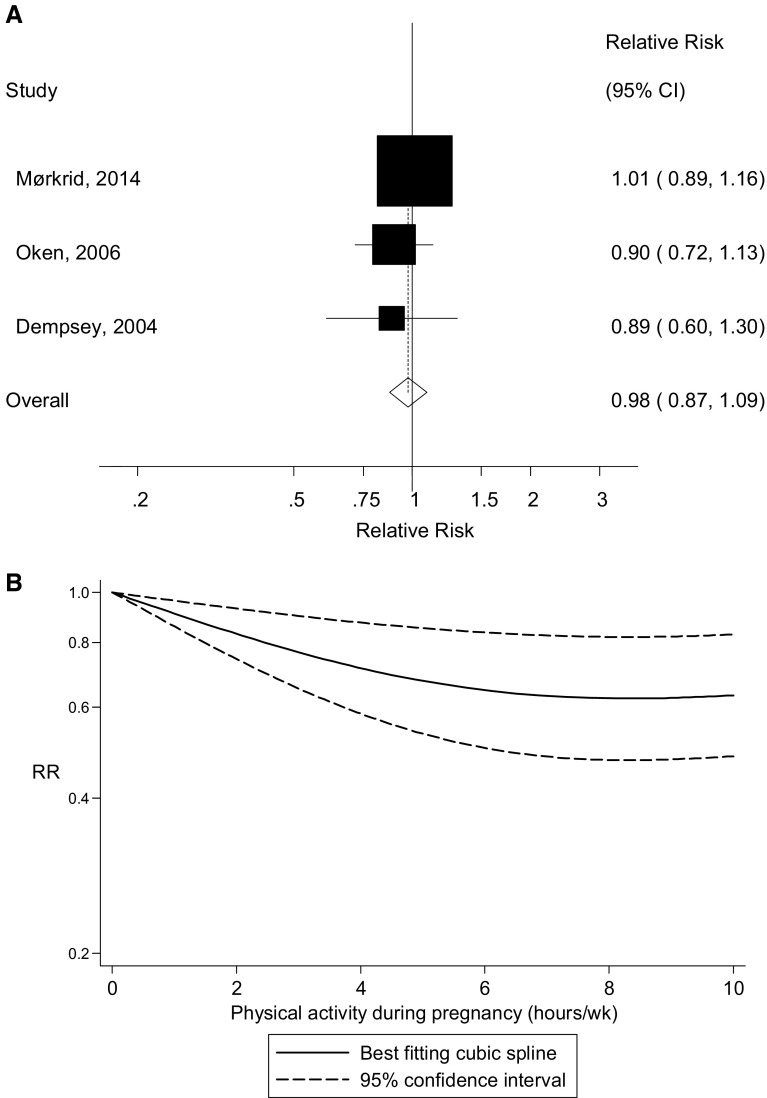
Leisure-time physical activity during early pregnancy and gestational diabetes mellitus, linear (per 5 h/week) and nonlinear dose–response analysis. **a** Leisure-time physical activity during pregnancy and gestational diabetes mellitus, per 5 h/week. **b** Leisure-time physical activity during pregnancy and gestational diabetes mellitus, nonlinear dose–response, h/week

Twelve randomized trials [11–22] and five cohort studies [23, 25, 27, 30, 33] were included in the analysis of early pregnancy physical activity and the risk of gestational diabetes mellitus and included 900 cases and 9804 participants. The summary RR for high versus low physical activity in early pregnancy was 0.80 (95 % CI 0.64–1.00, p_association_ = 0.046), with low heterogeneity, I^2^ = 17 %, p_heterogeneity_ = 0.26 (Fig. 5). The summary RR was 0.69 (95 % CI 0.50–0.96, I^2^ = 30.2 %, p_heterogeneity_ = 0.15) for the randomized trials and 0.97 (95 % CI 0.73–1.28, I^2^ = 0 %, p_heterogeneity_ = 0.80) for the cohort studies. There was evidence of publication bias with Egger’s test, *p* = 0.007. We also repeated the analysis using mid-pregnancy physical activity data instead of early pregnancy data for two studies [27, 33] which provided both, and the results were slightly strengthened, summary RR = 0.75 (95 % CI 0.59–0.95, I^2^ = 27.7 %, p_heterogeneity_ = 0.14). The summary RR per 5 h/week of physical activity was 0.98 (95 % CI 0.87–1.09, I^2^ = 0 %, p_heterogeneity_ = 0.59) (Fig. 6c) [23, 25, 34], but there was evidence of a nonlinear inverse association, p_nonlinearity_ = 0.008, with no further reduction in risk from approximately 7–8 h/week (Fig. 6d; Supplementary Table 2). Among the randomized trials we fitted a linear regression of the relative risks against the approximate total number of hours/week of the interventions, and although not statistically significant, *p* = 0.24, there was some indication of greater reductions in risk with a larger number of hours of activity (Supplementary Fig. 1). When the randomized trials were stratified by duration of activity among the studies for which we could estimate the approximate number of hours of activity per week the interventions lasted, the summary RR was 0.80 (95 % CI 0.37–1.71, *n* = 3) for studies with 1–2 h of activity per week, and 0.64 (0.44–0.93, *n* = 9) for studies with >2 h/week of activity, and 0.66 (95 % CI 0.44–1.01, *n* = 7) for >2–3 h/week, and 0.48 (95 % CI 0.18–1.27, *n* = 2) for >3 h/week.

### Combined pre-pregnancy and early pregnancy physical activity

**Fig. 7:**
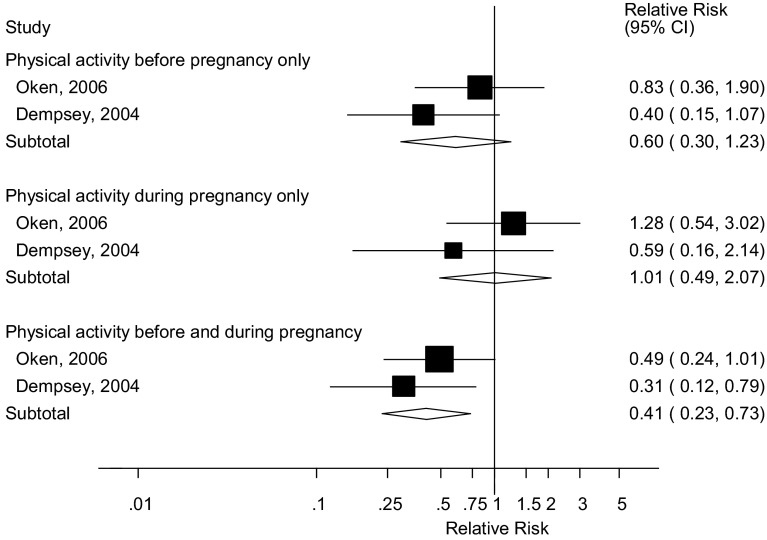
Leisure-time physical activity before and during pregnancy and gestational diabetes mellitus, joint associations

Two cohort studies [23, 25] investigated the association between combined physical activity before and during pregnancy and risk of gestational diabetes mellitus. The summary RR was 0.60 (95 % CI 0.30–1.23, I^2^ = 19.1 %, p_heterogeneity_ = 0.27) for physical activity before pregnancy only, 1.01 (95 % CI 0.49–2.07, I^2^ = 0 %, p_heterogeneity_ = 0.33) for physical activity during pregnancy only, and 0.41 (95 % CI 0.23–0.73, I^2^ = 0 %, p_heterogeneity_ = 0.45) for physical activity both before and during pregnancy (Fig. 7).

### Walking

**Fig. 8:**
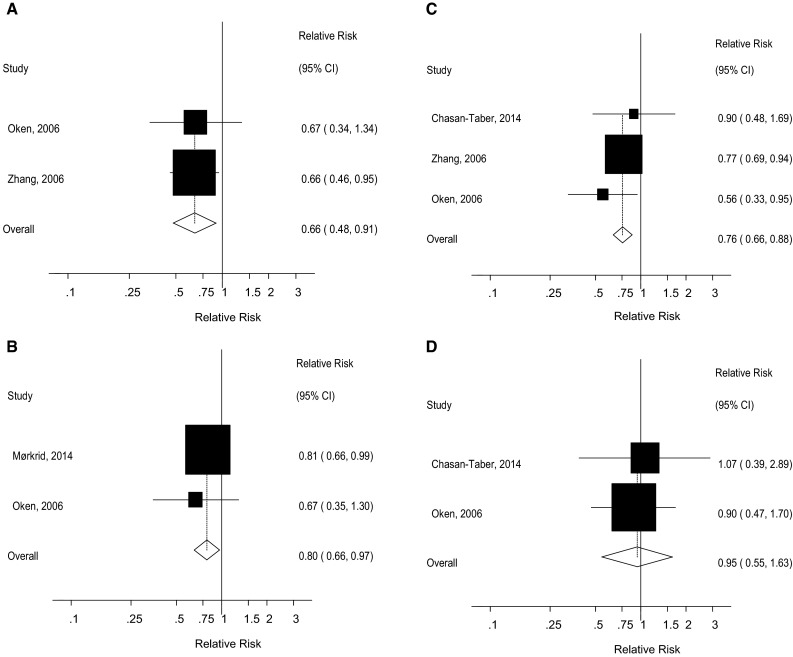
Walking and vigorous physical activity before and during pregnancy and gestational diabetes, high versus low comparison. **a** Walking before pregnancy and gestational diabetes mellitus, high versus low analysis. **b** Walking during pregnancy and gestational diabetes mellitus, high versus low analysis. **c** Vigorous physical activity before pregnancy and gestational diabetes mellitus, high versus low analysis. **d** Vigorous physical activity during pregnancy and gestational diabetes mellitus, high versus low analysis

Two cohort studies [24, 25] were included in the analysis of walking before pregnancy and gestational diabetes mellitus and two cohort studies [25, 30] were included in the analysis of walking during pregnancy and gestational diabetes mellitus. The summary RR was 0.66 (95 % CI 0.48–0.91, I^2^ = 0 %, p_heterogeneity_ = 0.97) for walking before pregnancy (Fig. 8a). The summary RR was 0.80 (95 % CI 0.66–0.97, I^2^ = 0 %, p_heterogeneity_ = 0.59) for walking during pregnancy (Fig. 8b).

### Intensity of physical activity

Three cohort studies [24, 25, 33] investigated the association between vigorous physical activity before pregnancy and gestational diabetes mellitus, while two cohort studies [25, 33] investigated vigorous physical activity in early pregnancy and gestational diabetes mellitus. The summary RR was 0.76 (95 % CI 0.66–0.88, I^2^ = 0 %, p_heterogeneity_ = 0.45) (Fig. 8c) for vigorous physical activity before pregnancy and 0.95 (95 % CI 0.55–1.63, I^2^ = 0 %, p_heterogeneity_ = 0.78) (Fig. 8d) for vigorous physical activity in early pregnancy.

### Occupational physical activity and household physical activity

Two cohort studies [27, 33] were included in the analysis of occupational physical activity before and during pregnancy. The summary RR for high versus low occupational physical activity was 1.90 (95 % CI 0.97–3.74, I^2^ = 0 %, p_heterogeneity_ = 0.79) for activity before pregnancy (Supplementary Fig. 2a) and 0.78 (95 % CI 0.21–2.93, I^2^ = 78 %, p_heterogeneity_ = 0.03) for activity during pregnancy (Supplementary Fig. 2b). Two cohort studies [27, 33] were included in the analysis of household physical activity before and during pregnancy. The summary RR for high versus low household physical activity was 0.36 (95 % CI 0.12–1.08, I^2^ = 61.9 %, p_heterogeneity_ = 0.11) for activity before pregnancy (Supplementary Fig. 3a) and 1.22 (95 % CI 0.53–2.81, I^2^ = 23.6 %, p_heterogeneity_ = 0.25) for activity during pregnancy (Supplementary Fig. 3b).

### Physical activity and abnormal glucose tolerance

We conducted supplementary analyses of one randomized trial [22] and five cohort studies [25, 33, 39, 44, 45] which reported on physical activity and abnormal glucose tolerance as an outcome and the summary RRs were 0.81 (95 % CI 0.55–1.17, I^2^ = 34 % p_heteroeneity_ = 0.21) for pre-pregnancy physical activity [25, 33, 39, 44] (Supplementary Fig. 4a) and 0.77 (95 % CI 0.63–0.95, I^2^ = 0 % p_heteroeneity_ = 0.76) for physical activity during pregnancy [22, 25, 33, 44, 45], respectively (Supplementary Fig. 4b).

### Subgroup, sensitivity, and meta-regression analyses

In subgroup and meta-regression analyses we found no significant heterogeneity between subgroups when studies were stratified by study design, geographic location, number of cases, study quality (cohort studies) or risk of bias (randomized trials) (Table 4). Further subgroup analyses by whether studies had adjusted for confounding factors did not reveal significant heterogeneity between most strata, although associations were not always statistically significant. We also conducted sensitivity analyses excluding one study at a time in the analyses of leisure-time physical activity, and although the summary relative risks did not vary substantially exclusion of some studies made the borderline significant associations statistically significant (Supplementary Figures 5, 6).

Mean (median) study quality scores were 7.0 (6.5) for cohort studies of leisure-time physical activity before pregnancy and 7.0 (7.0) for cohort studies of leisure-time physical activity during pregnancy. Of the 12 randomized trials 6 were deemed to be of high risk of bias, 1 of low risk of bias and 5 of unclear risk of bias.

We conducted further analyses of three studies on physical activity before pregnancy [23, 24, 27] and three studies on physical activity during pregnancy [23, 24, 27] and gestational diabetes mellitus which provided risk estimates adjusted and not adjusted for BMI, to clarify whether part of the association might be explained by reduced body fatness. The summary RR for pre-pregnancy physical activity was 0.63 (95 % CI 0.23–1.71) without BMI adjustment and 0.72 (95 % CI 0.30–1.76) with BMI adjustment, while for physical activity during pregnancy it was 0.49 (95 % CI 0.29–0.83) without BMI adjustment and 0.56 (95 % CI 0.33–0.95) with BMI adjustment.

## Discussion

In this meta-analysis higher leisure-time physical activity before and during pregnancy was associated with a marginally significant 22 % reduction and 20 % reduction in the relative risk of gestational diabetes mellitus, respectively. Higher total physical activity before pregnancy was associated with a 36 % reduction in the relative risk of gestational diabetes, while the association for total physical activity during pregnancy was in the direction of reduced risk, but was not statistically significant, possibly due to few studies. Walking before and during pregnancy and vigorous activity before pregnancy were also inversely associated with gestational diabetes, but occupational and household physical activity were not associated with risk, although these results were based on few studies.

When stratified by study design the association between leisure-time physical activity during pregnancy and gestational diabetes was significant in randomized trials, but not significant in cohort studies. As the studies differed with regard to the level of physical activity level between studies it is difficult to base physical activity recommendations on the results from the high versus low analyses, and therefore we also conducted linear and nonlinear dose–response analyses. In the nonlinear dose–response analysis there was a 12 % reduction in the relative risk of gestational diabetes mellitus for 30 MET-hours of pre-pregnancy physical activity per week compared to no activity, and a 30 % reduction in risk for 7 h of pre-pregnancy activity per week compared to no activity, and for physical activity during pregnancy there was a 37 % reduction in the relative risk for 7 h of activity per week compared to no activity. In analyses of combined pre-pregnancy and early pregnancy physical activity there was a suggestive 40 % reduction in risk among women who were physically active before pregnancy, but no association among women who were active only during early pregnancy, while there was a 59 % reduction in relative risk for women who were physically active before and during pregnancy compared to women who were inactive in both periods. An interesting finding of the present meta-analysis is that some of the associations were stronger for pre-pregnancy physical activity than for physical activity during pregnancy, which is similar to the findings in our meta-analysis on physical activity and preeclampsia [53]. This is not unreasonable as the time available to intervene, and the degree of physical activity that is achievable is more limited in pregnancy. In addition, the physiologic insulin-resistance in pregnancy could attenuate the effects of physical activity during pregnancy. However, as this was not entirely consistently observed across the various physical activity exposures, further studies with both prepregnancy and early pregnancy physical activity measures are needed for further clarification.

The results from this meta-analysis provide further support for the hypothesis that physical activity decreases the risk of gestational diabetes mellitus and are consistent with two previous meta-analyses which also found inverse association [41, 43], but not with a third [42]. However, in contrast to the previous meta-analyses we further quantified the association between physical activity and gestational diabetes mellitus risk by conducting linear and nonlinear dose–response analyses and conducted more detailed analyses of different domains of activity. Such analyses are important to guide recommendations to pregnant women with regard to the amount and types of physical activity that may reduce risk, as well as to inform future physical activity interventions that aim to reduce gestational diabetes risk.

Our meta-analysis may have some limitations that could have affected the results. It is possible that the observed inverse association between physical activity and risk of gestational diabetes mellitus risk could be due to unmeasured or residual confounding. Higher physical activity is associated with other healthy behaviors including lower prevalence of overweight and obesity and healthier diets with higher dietary fiber intake, and lower red and processed meat intake. However, many of the studies included in this meta-analysis adjusted for confounding factors such as age, BMI, and energy intake and the associations persisted in subgroup analyses by stratification by adjustment for these confounding variables. We found no evidence of heterogeneity between these subgroups with meta-regression analyses. Any further studies should adjust for more dietary confounding factors. There was moderate heterogeneity among studies of leisure-time activity before pregnancy (I^2^ = 47 %), but when stratified by number of cases there was no heterogeneity among studies with 200 or more cases (I^2^ = 0 %), although there was no between subgroup heterogeneity with meta-regression analyses. Among studies of leisure-time physical activity during pregnancy there was low heterogeneity (I^2^ = 17 %). For some other subtypes of activity there was high heterogeneity, but there were not enough studies to conduct subgroup and meta-regression analyses, or to test for publication bias and conduct other sensitivity analyses for the subtypes of physical activity. Not all the studies included in the high versus low analysis could be included in the dose–response analyses because results either were reported using a different underlying measure than others or because there was only a dichotomized categorization of physical activity. Any further studies should report the results for 3–4 or more categories of activity and use a measure that allows for combination with other studies, preferably in hours/week and/or MET-hours/week. In addition, some of the randomized trials may have had a dose of physical activity that was too low (1–2 h/week) to observe an association, particularly because of the possibility of contamination of the control group. In subgroup analyses of the randomized trials there was some suggestion of a stronger association among studies with an estimated duration of >2–3 and >3 h/week of activity than among those with an estimated duration of 1–2 h/week. In addition to the activity level being too low, the compliance with the exercise interventions may have been poor in some studies, which may have attenuated any associations. A challenge for future intervention studies will be to increase both the duration of the activity and the compliance with the interventions. In addition, much larger studies are needed as few of the randomized trials found statistically significant reductions in risk individually. As a meta-analysis of published studies publication bias may also have affected the results. There was evidence of publication bias in the analysis of leisure-time physical activity during pregnancy and risk of gestational diabetes, thus it is possible that this could have led to an exaggerated summary estimate.

Gestational diabetes and type 2 diabetes share many pathophysiological features and our previous findings of an inverse association between physical activity and type 2 diabetes [10] support the current results on gestational diabetes. Interestingly the strength and the shape of the dose–response relationships observed between leisure-time physical activity in relation to gestational diabetes and type 2 diabetes is similar with an approximate 20–30 % reduction in the relative risk observed with 5–7 h/week compared to no activity, and with a steeper reduction in the risk at lower levels of activity. Most likely several of the same mechanistic pathways are involved in explaining these results for gestational diabetes mellitus as for type 2 diabetes. Physical activity reduces adiposity [59] and has been associated with lower gestational weight gain [60–62], which is strongly related to increased risk of gestational diabetes mellitus [63]. Overweight and obesity increases inflammation, flux of free fatty acids, and may thereby lead to insulin resistance [64], which in turn increases the endogenous glucose production in the liver, while physical activity may counteract some of these adverse effects [25, 65]. In this analysis, we found that associations were 14–25 % weaker when adjusted for BMI compared with when not adjusted for BMI, suggesting that approximately 14–25 % of the association may be explained by reduced adiposity, and this is comparable with our previous meta-analysis on physical activity and type 2 diabetes where associations were 20–30 % weaker when adjusted for BMI compared with analyses not adjusted for BMI [10]. However, a clinically significant reduction remained after adjustment for BMI, suggesting that other mechanisms may be implicated. We also found a significant inverse association between pre-pregnancy physical activity and abnormal glucose tolerance. Physical activity increases glucose uptake and glycogen synthesis through increased glucose transport by the GLUT4 glucose transporters and increased activity of glycogen synthase [65]. Exercise increases the secretion of interleukin-6 (IL-6) from muscle cells, which has anti-inflammatory effects through inhibition of TNF-α and IL-1β, and reduces TNF-induced insulin resistance [65]. Physical activity has been associated with lower levels of total cholesterol, triglycerides, leptin, and improved glycemic control and reduced insulin resistance in pregnant women [25, 66–68]. The biological mechanism explaining the potential nonlinear association observed between physical activity and gestational diabetes mellitus is not clear and needs further study. In previous meta-analyses we have also observed similar nonlinear associations between physical activity and preeclampsia [53] and type 2 diabetes [10], with steeper reductions in risk at lower levels of activity, however, for all these three conditions reductions in risk have been observed up to between 5 and 7 h of activity per week. Given the similarities in the underlying pathophysiological features of preeclampsia, type 2 diabetes and gestational diabetes mellitus (e.g. insulin resistance, obesity), it is possible that some of the underlying mechanisms that may be common for all three conditions also partly could explain the nonlinearity. However, we can also not rule out the possibility that the nonlinearity partly could be due to few data points at higher levels of physical activity.

Our meta-analysis also has several strengths. Because we only included cohort studies and randomized trials, recall bias is not an issue and there is less potential for selection bias. We conducted dose–response analyses to investigate whether specific levels of physical activity were associated with gestational diabetes mellitus risk and found evidence of a dose–response relationship. Because of the increased sample size we had higher statistical power to detect an association than any individual study, however, most of the included studies had a moderate or small sample size. The quality of the cohort studies were in general high (mean scores of 7 out of 9 points), however, half of the randomized trials were at high risk of bias, and most of the remaining were at unclear risk of bias. Although, there was no heterogeneity by study quality scores or the risk of bias assessment when stratified, any future studies should improve the conduct and reporting of the results to provide better epidemiological evidence on physical activity and gestational diabetes mellitus.

Further large cohort studies and intervention trials are needed to conclusively establish the association between physical activity and specific types and intensities of physical activity and gestational diabetes, and for updated dose–response analyses it would be good if future studies could report results on a common scale, for example in hours/week and/or MET-hours/week. Any further intervention trials should aim to use a high enough dose or frequency of physical activity to be able to observe an effect (e.g. at least 2–3 h or more per week) and including multiple arms with different levels of physical activity might provide firm conclusions with regard to the dose–response relationship.

In conclusion, our results suggest that higher physical activity is associated with reduced risk of gestational diabetes mellitus. Any additional studies should assess the association between specific subtypes, amounts and intensities of physical activity and risk of gestational diabetes mellitus, adjust for more confounding factors and improve the reporting of the data.

## Electronic supplementary material

Below is the link to the electronic supplementary material.

Supplementary material 1 (DOC 64 kb)

Supplementary material 2 (DOCX 139 kb)
