# Supplementary material for: Physical activity and the risk of gestational diabetes mellitus: a systematic review and dose–response meta-analysis of epidemiological studies
Source: Eur J Epidemiol. 2016 Aug 2;31(10):967–97. doi: 10.1007/s10654-016-0176-0 (PMC5065594; doi:10.1007/s10654-016-0176-0)
Supplement: Supplementary file 2 — Supplementary material 2 (DOCX 139 kb) [file 10654_2016_176_MOESM2_ESM.docx]

Supplementary material for “Physical activity and the risk of gestational diabetes mellitus: a systematic review and dose-response meta-analysis of epidemiological studies”, by Dagfinn Aune, Abhijit Sen, Tore Henriksen, Ola Didrik Saugstad, Serena Tonstad.

Supplementary Table 1. List of excluded studies and exclusion reasons

| Exclusion reason | Reference number |
| --- | --- |
| Abstract | (1-7) |
| Case-control study | (8) |
| Combined lifestyle intervention | (9;10) |
| Commentary | (11) |
| Cross-sectional study | (12-20) |
| Duplicate | (21-24) |
| Gestational diabetes mellitus recurrence | (25) |
| Meta-analysis | (26-30) |
| No risk estimates | (31-36) |
| Not original data | (37-39) |
| Not relevant data | (40-43) |
| Not relevant exposure | (44-56) |
| Not relevant outcome | (57;58) |
| Protocol | (59;60) |
| Review | (61-87) |
| Unadjusted risk estimates | (88-90) |
| Yoga as intervention | (91) |

Reference List

1. Currie L, Woolcott C, Fell DF, Armson BA, Dodds L. Physical activity and pregnancy outcomes. American Journal of Epidemiology 2011;Conference:01.

2. Harrison CL, Lombard CB, Teede HJ. Understanding early pregnancy health behaviours among overweight and obese women at risk of gestational diabetes mellitus. Obesity Research and Clinical Practice 2011;Conference:October.

3. Van Poppel M, Oostdam N, Wouters M, Eekhoff M, Van MW. FitFor2: Effects of an exercise training program on the incidence of gestational diabetes. Journal of Science and Medicine in Sport 2012;Conference:December.

4. Mutsaerts MAQ, Groen H, Buiter-Van Der MA et al. Effects of paternal and maternal lifestyle factors on pregnancy complications and perinatal outcome. A Dutch population-based birth-cohort study: The GECKO Drenthe study. Human Reproduction 2012;Conference:2012.

5. El BN, Saad El DS, Mohamed R. Does physical activity and diet control reduce the risk of developing gestational diabetes mellitus in egypt? A randomized controlled trial. Journal of Perinatal Medicine 2013;Conference:June.

6. Hayes L, Bell R, Heslehurst N, Poston L. Low physical activity as a risk factor for gestational diabetes in obese white women and black women participating in the UK Better Eating and Activity Trial (UPBEAT). Pilot trial. Diabetic Medicine 2015;Conference:March.

7. Simmons D, Jelmsa J, Galjaard S et al. Results from a european multicentre, randomised trial of physical activity and/or healthy eating to reduce the risk of gestational diabetes mellitus (GDM): The dali pilot study. Diabetes Conference: 75th Scientific Sessions of the American Diabetes Association Boston, MA United States Conference Start: 2015;June.

8. Dempsey JC, Butler CL, Sorensen TK et al. A case-control study of maternal recreational physical activity and risk of gestational diabetes mellitus. Diabetes Res Clin Pract 2004;66:203-15.

9. Luoto R, Kinnunen TI, Aittasalo M et al. Primary prevention of gestational diabetes mellitus and large-for-gestational-age newborns by lifestyle counseling: a cluster-randomized controlled trial. PLoS Med 2011;8:e1001036.

10. Poston L, Briley AL, Barr S et al. Developing a complex intervention for diet and activity behaviour change in obese pregnant women (the UPBEAT trial); assessment of behavioural change and process evaluation in a pilot randomised controlled trial. BMC Pregnancy & Childbirth 13:148, 2013.

11. Ramirez-Velez R. A 12-week exercise program performed during the second trimester does not prevent gestational diabetes in healthy pregnant women. J Physiother 2012;58:198.

12. Seshiah V, Balaji V, Balaji MS et al. Prevalence of gestational diabetes mellitus in South India (Tamil Nadu)--a community based study. J Assoc Physicians India 2008;56:329-33.

13. Khatun N, Latif SA, Uddin MM. Risk factors for the development of gestational diabetes mellitus. Mymensingh Med J 2009;18:S20-S23.

14. Campbell SK, Lynch J, Esterman A, McDermott R. Pre-pregnancy predictors of diabetes in pregnancy among Aboriginal and Torres Strait Islander women in North Queensland, Australia. Matern Child Health J 2012;16:1284-92.

15. Anzaku AS, Musa J. Prevalence and associated risk factors for gestational diabetes in Jos, North-central, Nigeria. Arch Gynecol Obstet 2013;287:859-63.

16. Li Q, Xiong R, Wang L et al. Associations of dietary habits, physical activity and cognitive views with gestational diabetes mellitus among Chinese women. Public Health Nutr 2014;17:1850-7.

17. White E, Pivarnik J, Pfeiffer K. Resistance training during pregnancy and perinatal outcomes. J Phys Act Health 2014;11:1141-8.

18. Liu J, Laditka JN, Mayer-Davis EJ, Pate RR. Does physical activity during pregnancy reduce the risk of gestational diabetes among previously inactive women? Birth 2008;35:188-95.

19. Harizopoulou VC, Kritikos A, Papanikolaou Z et al. Maternal physical activity before and during early pregnancy as a risk factor for gestational diabetes mellitus. Acta Diabetol 2010;47 Suppl 1:83-9.

20. Redden SL, LaMonte MJ, Freudenheim JL, Rudra CB. The association between gestational diabetes mellitus and recreational physical activity. Matern Child Health J 2011;15:514-9.

21. Solomon CG, Willett WC, Carey VJ et al. A prospective study of pregravid determinants of gestational diabetes mellitus. Journal of the American Medical Association 1997;278:01.

22. Helseth R, Salvesen O, Stafne SN, Morkved S, Salvesen KA, Carlsen SM. Gestational diabetes mellitus among Nordic Caucasian women: Prevalence and risk factors according to WHO and simplified IADPSG criteria. Scandinavian Journal of Clinical and Laboratory Investigation 2014;74:01.

23. Rudra CB, Williams MA, Lee IM, Miller RS, Sorensen TK. Perceived exertion in physical activity and risk of gestational diabetes mellitus. Epidemiology 2006;17:31-7.

24. Ruiz JR, Perales M, Pelaez M, Lopez C, Lucia A, Barakat R. Supervised exercise-based intervention to prevent excessive gestational weight gain: A randomized controlled trial. Mayo Clinic Proceedings 2013;88:December.

25. Guelfi KJ, Ong MJ, Fournier PA et al. Does supervised home-based exercise during pregnancy reduce the recurrence of gestational diabetes? A randomised controlled trial. Journal of Paediatrics and Child Health Conference: 1919;April.

26. Tobias DK, Zhang C, Van Dam RM, Bowers K, Hu FB. Physical activity before and during pregnancy and risk of gestational diabetes mellitus: a meta-analysis. Diabetes Care 2011;34:223-9.

27. Yin YN, Li XL, Tao TJ, Luo BR, Liao SJ. Physical activity during pregnancy and the risk of gestational diabetes mellitus: a systematic review and meta-analysis of randomised controlled trials. Br J Sports Med 2014;48:290-5.

28. Russo LM, Nobles C, Ertel KA, Chasan-Taber L, Whitcomb BW. Physical Activity Interventions in Pregnancy and Risk of Gestational Diabetes Mellitus: A Systematic Review and Meta-analysis. Obstet Gynecol 2015;125:576-82.

29. Sanabria-Martinez G, Garcia-Hermoso A, Poyatos-Leon R, varez-Bueno C, Sanchez-Lopez M, Martinez-Vizcaino V. Effectiveness of physical activity interventions on preventing gestational diabetes mellitus and excessive maternal weight gain: A meta-analysis. BJOG: An International Journal of Obstetrics and Gynaecology 122 (9) (pp 1167-1174), 2015;01.

30. Bain E, Crane M, Tieu J, Han S, Crowther CA, Middleton P. Diet and exercise interventions for preventing gestational diabetes mellitus. Cochrane Database of Systematic Reviews 4:CD010443, 2015.

31. Dyck R, Klomp H, Tan LK, Turnell RW, Boctor MA. A comparison of rates, risk factors, and outcomes of gestational diabetes between aboriginal and non-aboriginal women in the Saskatoon health district. Diabetes Care 2002;25:487-93.

32. Retnakaran R, Qi Y, Sermer M, Connelly PW, Zinman B, Hanley AJG. Pre-gravid physical activity and reduced risk of glucose intolerance in pregnancy: The role of insulin sensitivity. Clinical Endocrinology 2009;70:April.

33. Harrison CL, Lombard CB, Teede HJ. Understanding health behaviours in a cohort of pregnant women at risk of gestational diabetes mellitus: an observational study. BJOG 2012;119:731-8.

34. Putnam KF, Mueller LA, Magann EF et al. Evaluating effects of self-reported domestic physical activity on pregnancy and neonatal outcomes in "stay at home" military wives. Mil Med 2013;178:893-8.

35. Hayes L, Bell R, Robson S, Poston L, UPBEAT Consortium. Association between physical activity in obese pregnant women and pregnancy outcomes: the UPBEAT pilot study. Annals of Nutrition & Metabolism 64(3-4):239-46, 2014.

36. Momeni JF, Simbar M, Dolatian M, Alavi MH. Comparison of lifestyles of women with gestational diabetes and healthy pregnant women. Glob J Health Sci 2015;7:162-9.

37. Dyck RF, Sheppard MS, Cassidy H, Chad K, Tan L, Van Vliet SH. Preventing NIDDM among aboriginal people: is exercise the answer? Description of a pilot project using exercise to prevent gestational diabetes. Int J Circumpolar Health 1998;57 Suppl 1:375-8.

38. Hegaard HK, Damm P, Nielsen BB, Pedersen BK. [Pregnancy and recreational physical activity]. Ugeskr Laeger 2006;168:564-6.

39. Zavorsky GS, Longo LD. Adding strength training, exercise intensity, and caloric expenditure to exercise guidelines in pregnancy. Obstet Gynecol 2011;117:1399-402.

40. Bell DS, Barger BO, Go RC et al. Risk factors for gestational diabetes in black population. Diabetes Care 1990;13:1196-201.

41. Kim C, McEwen LN, Kieffer EC, Herman WH, Piette JD. Self-efficacy, social support, and associations with physical activity and body mass index among women with histories of gestational diabetes mellitus. Diabetes Educ 2008;34:719-28.

42. Luoto RM, Kinnunen TI, Aittasalo M et al. Prevention of gestational diabetes: design of a cluster-randomized controlled trial and one-year follow-up. BMC Pregnancy Childbirth 2010;10:39.

43. Chasan-Taber L, Fortner RT, Gollenberg A, Buonnaccorsi J, Dole N, Markenson G. A prospective cohort study of modifiable risk factors for gestational diabetes among Hispanic women: design and baseline characteristics. J Womens Health (Larchmt ) 2010;19:117-24.

44. Berkowitz GS, Lapinski RH, Wein R, Lee D. Race/ethnicity and other risk factors for gestational diabetes. Am J Epidemiol 1992;135:965-73.

45. McMahon MJ, Ananth CV, Liston RM. Gestational diabetes mellitus. Risk factors, obstetric complications and infant outcomes. J Reprod Med 1998;43:372-8.

46. Xiong X, Saunders LD, Wang FL, Demianczuk NN. Gestational diabetes mellitus: prevalence, risk factors, maternal and infant outcomes. Int J Gynaecol Obstet 2001;75:221-8.

47. Cheung NW, Wasmer G, Al-Ali J. Risk factors for gestational diabetes among Asian women. Diabetes Care 2001;24:955-6.

48. Ogonowski J, Miazgowski T, Homa K, Celewicz Z, Kuczynska M. Low predictive value of traditional risk factors in identifying women at risk for gestational diabetes. Acta Obstet Gynecol Scand 2007;86:1165-70.

49. Yang H, Wei Y, Gao X et al. Risk factors for gestational diabetes mellitus in Chinese women: a prospective study of 16,286 pregnant women in China. Diabet Med 2009;26:1099-104.

50. Kun A, Tornoczky J, Tabak AG. The prevalence and predictors of gestational diabetes mellitus in Hungary. Hormone & Metabolic Research 2011;43:788-93.

51. Huy C, Loerbroks A, Hornemann A, Rohrig S, Schneider S. Prevalence, Trend and Determining Factors of Gestational Diabetes in Germany. Geburtshilfe Frauenheilkd 2012;72:311-5.

52. Reeske A, Zeeb H, Razum O, Spallek J. Differences in the Incidence of Gestational Diabetes between Women of Turkish and German Origin: An Analysis of Health Insurance Data From a Statutory Health Insurance in Berlin, Germany (AOK), 2005-2007. Geburtshilfe Frauenheilkd 2012;72:305-10.

53. Egerman R, Ramsey R, Istwan N, Rhea D, Stanziano G. Maternal characteristics influencing the development of gestational diabetes in obese women receiving 17- alpha -hydroxyprogesterone caproate. Journal of Obesity 2014;2014.

54. AlKasseh AS, Zaki NM, Aljeesh YI, Soon LK. Risk factors of gestational diabetes mellitus in the refugee population in Gaza Strip: a case-control study. East Mediterr Health J 2014;19 Suppl 3:S12-S18.

55. Rajput M, Bairwa M, Rajput R. Prevalence of gestational diabetes mellitus in rural Haryana: A community-based study. Indian J Endocrinol Metab 2014;18:350-4.

56. Merriam AA, Chichester M, Patel N, Hoffman MK. Bed rest and gestational diabetes: more reasons to get out of bed in the morning. Obstet Gynecol 2014;123 Suppl 1:70S.

57. Korpi-Hyovalti E, Heinonen S, Schwab U, Laaksonen DE, Niskanen L. Effect of intensive counselling on physical activity in pregnant women at high risk for gestational diabetes mellitus. A clinical study in primary care. Prim Care Diabetes 2012;6:261-8.

58. McIntyre HD, Peacock A, Miller YD, Koh D, Marshall AL. Pilot study of an individualised early postpartum intervention to increase physical activity in women with previous gestational diabetes. Int J Endocrinol 2012;2012:892019.

59. Chasan-Taber L, Marcus BH, Stanek E, III et al. A randomized controlled trial of prenatal physical activity to prevent gestational diabetes: design and methods. J Womens Health (Larchmt ) 2009;18:851-9.

60. Nagle C, Skouteris H, Morris H et al. Primary prevention of gestational diabetes for women who are overweight and obese: A randomised controlled trial. BMC Pregnancy and Childbirth 2013;13.

61. Ruderman N, Apelian AZ, Schneider SH. Exercise in therapy and prevention of type II diabetes. Implications for blacks. Diabetes Care 1990;13:1163-8.

62. Sternfeld B. Physical activity and pregnancy outcome. Review and recommendations. Sports Med 1997;23:33-47.

63. Brown W. The benefits of physical activity during pregnancy. J Sci Med Sport 2002;5:37-45.

64. Dempsey JC, Butler CL, Williams MA. No need for a pregnant pause: physical activity may reduce the occurrence of gestational diabetes mellitus and preeclampsia. Exerc Sport Sci Rev 2005;33:141-9.

65. Weissgerber TL, Wolfe LA, Davies GAL, Mottola MF. Exercise in the prevention and treatment of maternal-fetal disease: A review of the literature. Applied Physiology, Nutrition and Metabolism 2006;31:December.

66. Ceysens G, Rouiller D, Boulvain M. Exercise for diabetic pregnant women. Cochrane database of systematic reviews (Online) 2006;3:2006.

67. Impact of physical activity during pregnancy and postpartum on chronic disease risk. Med Sci Sports Exerc 2006;38:989-1006.

68. Brown WJ, Burton NW, Rowan PJ. Updating the Evidence on Physical Activity and Health in Women. American Journal of Preventive Medicine 2007;33:November.

69. Mottola MF. The role of exercise in the prevention and treatment of gestational diabetes mellitus. Curr Sports Med Rep 2007;6:381-6.

70. Hegaard HK, Pedersen BK, Nielsen BB, Damm P. Leisure time physical activity during pregnancy and impact on gestational diabetes mellitus, pre-eclampsia, preterm delivery and birth weight: a review. Acta Obstet Gynecol Scand 2007;86:1290-6.

71. Dode MA, dos S, I. Non classical risk factors for gestational diabetes mellitus: a systematic review of the literature. Cad Saude Publica 2009;25 Suppl 3:S341-S359.

72. Melzer K, Schutz Y, Boulvain M, Kayser B. Physical activity and pregnancy: cardiovascular adaptations, recommendations and pregnancy outcomes. Sports Med 2010;40:493-507.

73. Oostdam N, van Poppel MN, Wouters MG, Van MW. Interventions for preventing gestational diabetes mellitus: a systematic review and meta-analysis. J Womens Health (Larchmt ) 2011;20:1551-63.

74. Zhang C, Ning Y. Effect of dietary and lifestyle factors on the risk of gestational diabetes: review of epidemiologic evidence. Am J Clin Nutr 2011;94:1975S-9S.

75. Oteng-Ntim E, Varma R, Croker H, Poston L, Doyle P. Lifestyle interventions for overweight and obese pregnant women to improve pregnancy outcome: Systematic review and meta-analysis. BMC Medicine 2012;10.

76. Han S, Middleton P, Crowther CA. Exercise for pregnant women for preventing gestational diabetes mellitus. Cochrane Database Syst Rev 2012;7:CD009021.

77. Thangaratinam S, Rogozinska E, Jolly K et al. Effects of interventions in pregnancy on maternal weight and obstetric outcomes: meta-analysis of randomised evidence. BMJ 2012;344:e2088.

78. Chasan-Taber L. Physical activity and dietary behaviors associated with weight gain and impaired glucose tolerance among pregnant Latinas. Adv Nutr 2012;3:108-18.

79. Ferraro ZM, Gaudet L, Adamo KB. The potential impact of physical activity during pregnancy on maternal and neonatal outcomes. Obstet Gynecol Surv 2012;67:99-110.

80. Colberg SR, Castorino K, Jovanovic L. Prescribing physical activity to prevent and manage gestational diabetes. World Journal of Diabetes 2013;4:December.

81. Hopkins SA, Artal R. The role of exercise in reducing the risks of gestational diabetes mellitus. Womens Health (Lond Engl ) 2013;9:569-81.

82. Ruchat SM, Mottola MF. The important role of physical activity in the prevention and management of gestational diabetes mellitus. Diabetes Metab Res Rev 2013;29:334-46.

83. SKlempe K, I, Ivanisevic M. Preventive effects of exercise on the occurrence of gestational diabetes mellitus. [Croatian]. Gynaecologia et Perinatologia 2013;22:2013.

84. Kokic IS. Exercise and gestational diabetes mellitus. Periodicum Biologorum 2014;116:March.

85. van Poppel MN, Ruchat SM, Mottola MF. Physical activity and gestational diabetes mellitus. Med Sport Sci 2014;60:104-12.

86. Artal R. The role of exercise in reducing the risks of gestational diabetes mellitus in obese women. Best Pract Res Clin Obstet Gynaecol 2015;29:123-32.

87. Feig DS. Physical activity during pregnancy: Is it wise? Canadian Journal of Diabetes 2012;36:2012.

88. Dye TD, Knox KL, Artal R, Aubry RH, Wojtowycz MA. Physical activity, obesity, and diabetes in pregnancy. Am J Epidemiol 1997;146:961-5.

89. Dode MA, Santos IS. [Risk factors for gestational diabetes mellitus in the birth cohort in Pelotas, Rio Grande do Sul State, Brazil, 2004]. Cad Saude Publica 2009;25:1141-52.

90. Mutsaerts MAQ, Groen H, Buiter-Van Der MA et al. Effects of paternal and maternal lifestyle factors on pregnancy complications and perinatal outcome. A population-based birth-cohort study: The GECKO Drenthe cohort. Human Reproduction 2014;29:2014.

91. Rakhshani A, Nagarathna R, Mhaskar R, Mhaskar A, Thomas A, Gunasheela S. The effects of yoga in prevention of pregnancy complications in high-risk pregnancies: A randomized controlled trial. Preventive Medicine 2012;55:October.

Supplementary Table 2. Relative risks and 95% confidence intervals from the nonlinear dose-response analysis

| Physical activity before pregnancy | | | | Physical activity during pregnancy | | | |
| --- | --- | --- | --- | --- | --- | --- | --- |
| MET-hours/week | Summary RR (95% CI) | Hours/week | Summary RR (95% CI) | MET-hours/week | Summary RR (95% CI) | Hours/week | Summary RR (95% CI) |
| 0 | 1.00 | 0 | 1.00 | 0 | - | 0 | 1.00 |
| 5 | 0.97 (0.95-1.00) | 1 | 0.92 (0.90-0.95) | 5 | - | 1 | 0.91 (0.86-0.96) |
| 10 | 0.94 (0.90-0.99) | 2 | 0.86 (0.82-0.90) | 10 | - | 2 | 0.83 (0.74-0.93) |
| 15 | 0.92 (0.86-0.99) | 3 | 0.81 (0.76-0.86) | 15 | - | 3 | 0.77 (0.65-0.90) |
| 20 | 0.90 (0.84-0.97) | 4 | 0.77 (0.71-0.83) | 20 | - | 4 | 0.71 (0.58-0.87) |
| 25 | 0.89 (0.83-0.96) | 5 | 0.74 (0.68-0.80) | 25 | - | 5 | 0.67 (0.53-0.85) |
| 30 | 0.88 (0.82-0.95) | 6 | 0.72 (0.65-0.78) | 30 | - | 6 | 0.65 (0.50-0.84) |
| 35 | - | 7 | 0.70 (0.63-0.77) | 35 | - | 7 | 0.63 (0.48-0.83) |
| 40 | - | 8 | 0.68 (0.61-0.77) | 40 | - | 8 | 0.62 (0.47-0.82) |
| 45 | - | 9 | 0.67 (0.59-0.77) | 45 | - | 9 | 0.63 (0.48-0.82) |
| 50 | - | 10 | 0.66 (0.57-0.77) | 50 | - | 10 | 0.63 (0.48-0.83) |
| p_nonlinearity_ | 0.31 | p_nonlinearity_ | 0.005 | p_nonlinearity_ | - | p_nonlinearity_ | 0.008 |

MET=metabolic equivalent task, 95% CI=95% confidence interval, RR=relative risk

Supplementary Figure 1. Physical activity during pregnancy and gestational diabetes mellitus, linear regression of RRs against number of hours of physical activity per week (randomized controlled trials)

Supplementary Figure 2. Occupational physical activity before and during pregnancy and gestational diabetes mellitus

Supplementary Figure 3. Household physical activity before and during pregnancy and gestational diabetes mellitus, high vs. low comparison

Supplementary Figure 4. Leisure-time physical activity before and during pregnancy and abnormal glucose tolerance, high vs. low comparison

Supplementary Figure 5. Influence analysis of leisure-time physical activity before pregnancy and gestational diabetes mellitus

------------------------------------------------------------------------------

Study omitted | e^coef. [95% Conf. Interval]

-------------------+----------------------------------------------------------

Chasan-Taber, 2014| 0.75306171 0.58002573 0.97771859

Mørkrid, 2014 | 0.81521887 0.60065728 1.10642420

Ramos-Levi, 2012 | 0.80838400 0.59478199 1.09869620

van der Ploeg, 2011| 0.72992229 0.56582469 0.94161075

Chasan-Taber, 2008| 0.75097895 0.59148514 0.95348018

Oken, 2006 | 0.78646427 0.59745240 1.03527260

Zhang, 2006 | 0.77771908 0.54743731 1.10487000

Dempsey, 2004 | 0.80902970 0.66491061 0.98438656

-------------------+----------------------------------------------------------

Combined | 0.77843730 0.60578869 1.00029040

------------------------------------------------------------------------------

Supplementary Figure 6. Influence analysis of leisure-time physical activity during pregnancy and gestational diabetes mellitus

------------------------------------------------------------------------------

Study omitted | e^coef. [95% Conf. Interval]

-------------------+----------------------------------------------------------

Nobles, 2015 | 0.80677223 0.6409936 1.0154257

Ko, 2014 | 0.79102534 0.61764884 1.0130694

Cordero, 2014 | 0.84548444 0.70029384 1.020777

Renault, 2014 | 0.82890069 0.67256629 1.0215741

Barakat, 2014 | 0.79107594 0.6273042 0.99760389

Tomic, 2013 | 0.8647396 0.7178638 1.0416664

Barakat, 2013 | 0.7820729 0.61060351 1.0016942

Barakat, 2012 | 0.81777847 0.66203392 1.0101622

Oostdam, 2012 | 0.80330664 0.63663685 1.0136102

Stafne, 2012 | 0.76652819 0.61089039 0.96181816

Price, 2011 | 0.79399985 0.63046467 0.9999541

Callaway, 2010 | 0.78354144 0.62585562 0.98095661

Chasan-Taber, 2014| 0.77776909 0.61953026 0.97642481

Mørkrid, 2014 | 0.75427967 0.59274691 0.95983249

Chasan-Taber, 2008| 0.79603863 0.63214052 1.0024314

Oken, 2006 | 0.78579313 0.62136036 0.99374032

Dempsey, 2004 | 0.80348784 0.635391 1.0160558

-------------------+----------------------------------------------------------

Combined | 0.80038761 0.64325167 0.99590932

------------------------------------------------------------------------------
